# Exploring the Presence of Cannabinoids in Hemp-Infused Fermented Milk Drinks: An Analysis of Pre- and Post-Fermentation Levels

**DOI:** 10.3390/molecules29215056

**Published:** 2024-10-26

**Authors:** Joanna Kanabus, Marcin Bryła, Katarzyna Kycia, Joanna Markowska, Marek Roszko

**Affiliations:** 1 Department of Food Safety and Chemical Analysis, Institute of Agricultural and Food Biotechnology—State Research Institute, Rakowiecka 36, 02-532 Warsaw, Poland; marcin.bryla@ibprs.pl (M.B.); marek.roszko@ibprs.pl (M.R.); 2 Inter-Department Problem Group for Diary Industries, Institute of Agricultural and Food Biotechnology—State Research Institute, Rakowiecka 36, 02-532 Warsaw, Poland; katarzyna.kycia@ibprs.pl; 3 Department of Refrigeration Technology and Technique, Institute of Agricultural and Food Biotechnology—State Research Institute, Al. Marszalka J. Pilsudskiego 84, 92-202 Lodz, Poland; joanna.markowska@ibprs.pl

**Keywords:** cannabinoids, fermentation, fermented milk drinks, hemp oil, dried hemp, hemp extract

## Abstract

Yoghurts are the most popular fermented dairy products. Consumer attention is directed towards products containing inputs that enrich the product with bioactive substances. The growing interest in the *Cannabis sativa* L. plant has resulted in the development of a market for hemp products. The main aim of this research work was to determine the effect of the lactic fermentation process on the stability of cannabinoids in fermented milk beverages containing hemp inputs (hemp oil, dried hemp, ethanolic hemp extract) at 0.5, 1 and 2% (*w*/*v*). The effect of the type of hemp input on the technological process (i.e., pH value, viability of LAB during 28 days of storage) was also checked and the sensory quality of the prepared samples was evaluated. It was shown that the choice of type/form and amount of hemp addition influenced the final cannabinoid content of the product after fermentation and post-fermentation, but no effect on the survival of yoghurt bacteria or pH changes during storage was confirmed. Fermented milk drinks containing hemp oil had the highest cannabinoid content. The QDA results of the sensory evaluation of the yoghurts show that the most acceptable product in terms of overall quality is the yoghurt containing 0.5% hemp extract and 2% hemp oil.

## 1. Introduction

Yoghurt is one of the leading dairy products. According to Codex Alimentarius [1], yoghurt is defined as fermented milk with symbiotic starter cultures of Streptococcus thermophilus and *Lactobacillus delbrueckii subsp. bulgaricus*. The yoghurt bacteria (probiotics) must be present in the yoghurt at a minimum of 10^7^ CFU/mL throughout the shelf life, and manufacturers must declare their presence on the label. Probiotics are ‘live microorganisms that confer health benefits to the host when administered adequately’ [2]. The quality of this fermented dairy beverage is primarily determined by its physical properties, i.e., texture, stability (rate of syneresis) and appropriate consistency [3]. The aforementioned physical characteristics of yoghurt are primarily influenced by the conditions of the fermentation process (e.g., heat treatment of the milk, time and temperature of the process, and cooling time and rate). The quality of yoghurt is undoubtedly influenced by the ingredients used (e.g., milk proteins, stabilisers, fibre and prebiotics), which are usually used for technological, functional or nutritional reasons [3,4,5].

Functional foods have been proven to have a beneficial effect on one or more bodily functions over and above the nutritional impact, which effect amounts to an improvement in health and well-being and a reduction in the risk of disease [6,7]. These foods are distinguished by their content of bioactive substances, which stimulate the desired metabolic pathway. In contrast, according to Świderski and Kolanowski [8], functional foods should be separated from functional additives. Substances added to food can be divided into two groups: food additives used in the technological process, which directly or indirectly can become food ingredients, and substances added to preserve or improve the nutritional value [7]. These include, among other things, texture stabilisers (agar, inulin, carrageenan, pectin), flavourings (fruit and vegetable injections, cereal grains, nuts, plant extracts), as well as substances of plant origin containing bioactive substances (caffeine, fibre). One of the plant-based additives may be dried *Cannabis sativa* L. *var. sativa* plant, as well as extracts or oils of hemp, which contain cannabinoids. This group of compounds has proven health-promoting properties for the human body (e.g., anticonvulsant, anxiolytic and anti-rheumatoid arthritis properties), among which there are two main compounds, i.e., the non-psychoactive CBD (cannabidiol) and the psychoactive Δ^9^-THC (Δ^9^-tetrahydrocannabinol) [9]. The positive aspects of adding cannabinoids to food in different charge forms is that the nutritional value of the prepared product can be improved. By introducing cannabinoids dissolved in oil, we also provide omega-3 and omega-6 unsaturated fatty acids, which support the human body’s function. The use of dried hemp allows us to increase the amount of protein in the yoghurt and fibre, which also play an important role in human nutrition [9,10]. The development of a hemp extract product with a specific CBD content requires adequate research and knowledge of the raw material. The resulting dried/extract quality depends on the plant variety, growing conditions, harvest time and extraction methods. Cannabinoids are chemically transformed over time by heating, oxidation and interaction with other food components, including enzymes. Therefore, it is essential to estimate the degree of degradation of active ingredients in the food matrix and the product’s suitability for consumption [10].

The main aim of this study was to evaluate the influence of the fermentation process and storage time on changes in the cannabinoid profile of model fermented milk drinks with hemp inputs (hemp oil, dried hemp, ethanolic hemp extract) at 0.5, 1 and 2% (*w*/*v*). Also, the effect of the hemp addition to the fermented milk drink on changes in pH and lactic acid bacteria viability before and after fermentation and during 28 days of storage was evaluated. In addition, the sensory quality and overall quality of the prepared beverages were also assessed. This is the first paper focusing on preparing fermented dairy beverages with hemp inputs and analysing selected parameters, including the cannabinoid content of the prepared samples.

## 2. Results and Discussion

### 2.1. Determination of Cannabinoids in Fermented Milk Drink Samples

**Figure 1:**
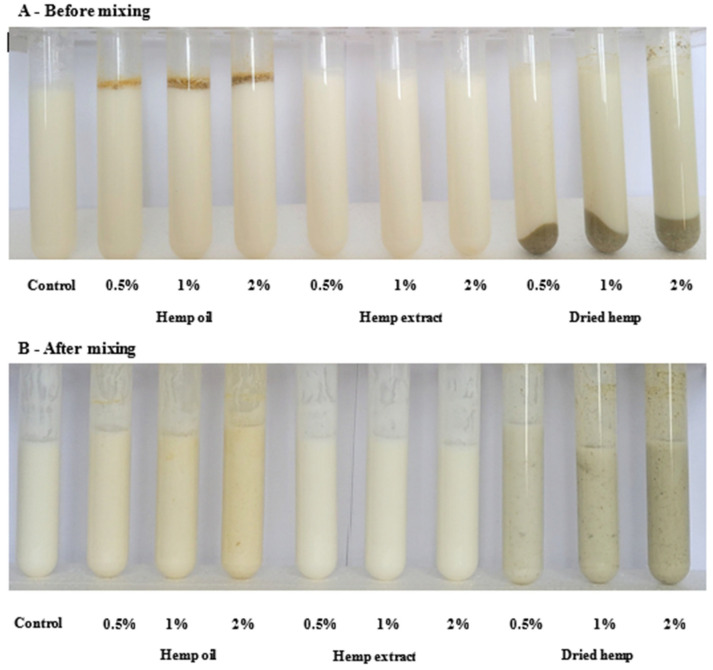
Finished fermented milk drink before (**A**) and after mixing (**B**) (own preparation).

The main aim of this study was to evaluate changes in the cannabinoid profile during the production of a fermented dairy beverage by adding different hemp inputs in three variants of additive amounts (0.5, 1 and 2% (*w*/*v*)). Figure 1 shows the finished fermented milk drinks with hemp inputs before (A) and after mixing (B).

**Figure 2:**
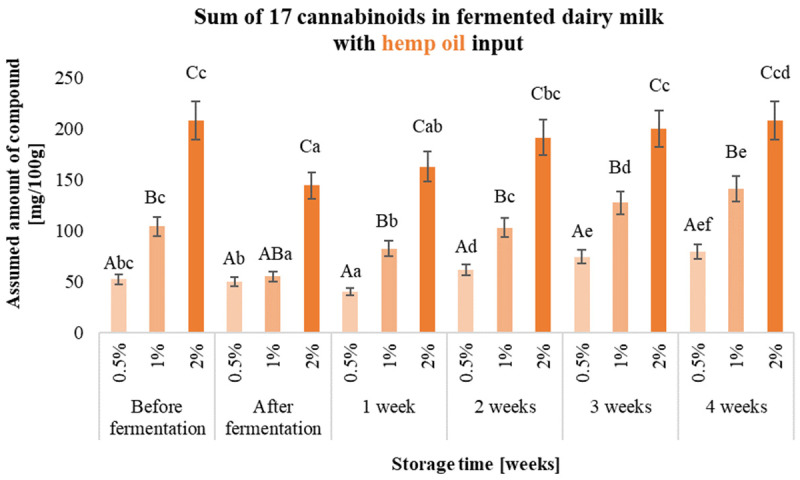
Sum of 17 cannabinoids in fermented dairy milk with hemp oil inputs (0.5%, 1% and 2% (*w*/*v*)) before and after fermentation and during 4 weeks of storage. ^a–f^—the different small letters in one amount of the input used, for example, 0.5%, indicate a significant difference (α < 0.05) influenced by storage time; ^A–C^—the different capital letters within the different samples during a specific period of production and storage, e.g., ‘after fermentation’, indicate significant differences (α < 0.05).

**Figure 3:**
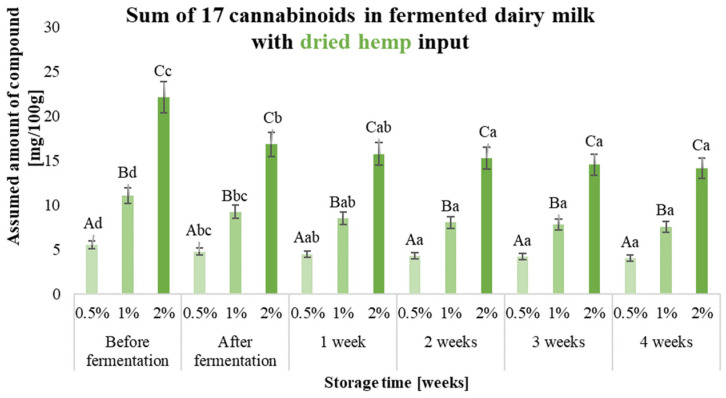
Sum of 17 cannabinoids in fermented dairy milk with dried hemp inputs (0.5%, 1% and 2% (*w*/*v*)) before and after fermentation and during 4 weeks of storage. ^a–d^—the different small letters in one amount of the input used, for example, 0.5%, indicate a significant difference (α < 0.05) influenced by storage time; ^A–C^—the different capital letters within the different samples during a specific period of production and storage, e.g., ‘after fermentation’, indicate significant differences (α < 0.05).

**Figure 4:**
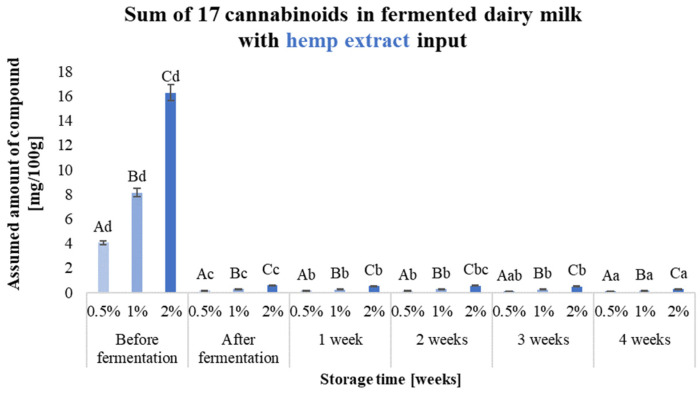
Sum of 17 cannabinoids in fermented dairy milk with hemp extract inputs (0.5%, 1% and 2% (*w*/*v*)) before and after fermentation and during 4 weeks of storage. ^a–d^—the different small letters in one amount of the input used, for example, 0.5%, indicate significant differences (α < 0.05) influenced by storage time; ^A–C^—the different capital letters within the different samples during a specific period of production and storage, e.g., ‘after fermentation’, indicate significant differences (α < 0.05).

Validation of the method for the determination of cannabinoids in hemp fermented milk drinks was carried out, and the results (LOD—limit of detection, LOQ—limit of quantitation, R%—recovery rate and RDS%—method repeatability) are presented in Tables S1 and S2 (Supplementary Materials). The effect of storing the prepared samples before 28 days under refrigeration (4 °C) was also checked, and samples for analysis were taken every 7 days. The results obtained for the 17 cannabinoids analysed are shown in Figure 2, Figure 3 and Figure 4 and Tables S3–S5 (Supplementary Materials).

Different forms of hemp additive resulted in the production of fermented milk drinks characterised by varying levels of cannabinoids. The highest initial total of 17 cannabinoids was characterised by beverages containing a hemp oil-based input (e.g., 1%—103 mg/100 g of product), while the least amount of these compounds was contained in beverages with hemp extract added (e.g., 1%—8.14 mg/100 g of product). Carrying out the fermentation process of dairy beverages with the addition of hemp extract significantly contributed to a more than 10-fold degradation of the cannabinoids present in the product.

In fermented milk drinks containing dried hemp and with the addition of 0.5% hemp oil, no significant reduction in total cannabinoids was observed after the fermentation process. The use of hemp inputs in the form of hemp oil or dried hemp provides up to 10 times the concentration of cannabinoids that are naturally present in the plant or allows more compounds to be concentrated by dissolving them in oil. Cannabinoids are compounds readily soluble in fat, which protects these compounds by preventing their degradation through, for example, oxidation or the heating necessary for pasteurisation and fermentation. The compounds in dried hemp are also not directly exposed to temperature or oxygen, as they are located in the glandular trichomes, where they are synthesised and stored in the plant. The choice of a feedstock in the form of an ethanolic hemp extract does not guarantee a protective effect on the bioactive substances extracted from the plant. Selecting a suitable extraction solvent is important, e.g., herbal extracts. For such extracts, it is possible to use water or ethyl alcohol. Other solvents, i.e., hexane or dichloromethane, are not allowed, and their potential use for extraction requires the solvent to be removed and such a product to be subjected to in vitro and in vivo testing to exclude toxicity [11]. Consumer expectations give direction to yoghurt producers, which should focus on the quantity and variety of products with unique added value.

By exposing the prepared samples of fermented dairy drinks to storage, the stability of both the sum of the 17 analysed cannabinoids and the individual compounds could be determined. The use of inputs in the form of hemp oil yielded products which, after 4 weeks of storage, were characterised by a comparable or higher sum of cannabinoids in the beverage. The cannabinoid content of milk beverages containing dried hemp oil during storage did not change significantly in any variants. The sum of 17 cannabinoids in samples containing ethanolic hemp extract at the beginning of storage was considerably higher than after storage in all variants, where it decreased by more than 90%. The fermentation process significantly affected cannabinoid degradation in the cannabinoid extract product. The high degradation of these compounds (compared to other inputs) may have been due to the absence of structure-stabilising factors for the cannabinoid molecule, which are present in the hemp oil-based and dried inputs [12]. The predominant cannabinoids in the hemp oil-based inputs were CBD (1% input—42.57 mg/100 g of product) and CBG (1% input—51.11 mg/100 g of product), while CBDA was the predominant cannabinoid in the hemp-dried and hemp extract-based inputs (for 1% input—5.32 mg and 3.75 mg/100 g of product, respectively) (Tables S3–S5).

The concentration of CBD increased up to 2-fold after fermentation and storage of the samples containing the hemp oil-based batch, depending on the amount of additive. In the case of CBG, the concentration of this compound, depending on the amount of additive, decreased by up to 10-fold after fermentation, and a gradual increase in concentration was observed during storage, but did not reach the level for the fresh product (milk with the batch before fermentation). The increase in the concentration of CBD and the decrease in the amount of other compounds may be due to, for example, decarboxylation of the acidic precursors for this compound and also the possible isomerisation of Δ9-THC to CBD [9]. In samples with ethanolic hemp extract added, the fermentation process resulted in more than 90% degradation of CBDA, the concentration of which also decreased during storage.

After fermentation, no cannabinoids were observed in the hemp extract samples analysed, i.e., CBC, CBG, CBDV, Δ^9^-THCV, Δ^9^-THCVA, CBN, CBNA, CBL and CBLA. No Δ^8^-THC was found in any additive variants, and the production process of the fermented dairy beverage with each hemp input also did not contribute to this compound. During storage, CBL and CBLA were not observed in yoghurt samples containing dried hemp. The use of hemp-based batches also carries a risk of the psychoactive Δ^9^-THC and its precursor Δ^9^-THCA. As a result of the fermentation process, more than 90% degradation of these compounds was observed in all variants of fermented milk drinks. Refrigerated storage for 28 days did not affect changes in the concentrations of the compounds in question. Despite the potential risk of Δ^9^-THC in products containing hemp inputs, it has been confirmed that, when delivered to the body via the oral route, both Δ^9^-THC and CBD are degraded in the acidic environment of the stomach and are broken down by enzymes in the gut, resulting in a bioavailability of <20% [13,14]. The observed decrease in Δ^9^-THC and Δ^9^-THCA concentrations was probably caused by the effect of temperature during the pasteurisation of milk with hemp inputs and the impact of oxygen [15,16].

In 2015, the European Food Safety Authority (EFSA) published a scientific opinion that an adult human’s acute reference dose for total Δ^9^-THC should be 1 μg Δ^9^-THC/kg body weight [17]. In contrast, an in-depth European Industrial Hemp Association (EIHA) analysis suggests that this value is too stringent and should be increased to 7 μg Δ^9^-THC/kg body weight. A total daily intake of 5 mg Δ^9^-THC results in a LOEL (Lowest Observable Adverse Effect Level) of 0.07 mg Δ^9^-THC/kg body weight (b.w.) per day, assuming a body weight of 70 kg [18]. Based on this, it can be assumed that the average daily intake of total Δ^9^-THC for a person weighing 70 kg is 70 μg according to the EFSA and 490 μg according to the EIHA. The following equation can be used to assess the potential risk of Δ^9^-THC being present at too high a level in the resulting finished product: the amount of Δ^9^-THC + (0.877 × amount of Δ^9^-THCA-A) [19]. A comparison of the assumptions presented and the results obtained for the prepared model hemp fermented milk drinks are shown in Table 1.

Based on the total Δ^9^-THC content results obtained for the samples containing the hemp inputs, it can be concluded that the finished product after fermentation and after 28 days of refrigerated storage is safe for a potential consumer weighing 70 kg and consuming 100 g of finished product per day.

### 2.2. Viability of Yoghurt Cultures

Changes in the number of viable cells of *S. thermophilus* and *Lactobacillus delbrueckii* ssp. *bulgaricus* after fermentation and during 1, 7, 14, 21 and 28 days of storage at 4 °C are shown in Table 2.

The initial number of live *S. thermphilus* cells after fermentation ranged from 10.0 to 8.7 log cfu/mL and decreased slightly after 28 days of refrigerated storage and ranged from 9.3 to 8.1 log CFU/mL. The greatest decrease in the number of live *S. thermophilus* bacteria was recorded for the sample containing 1% ethanolic hemp extract, while the number of bacteria increased slightly in the other variants of this additive. The use of three different hemp batches did not adversely affect the number of *S. thermophilus* bacteria during cold storage, and at the end of storage, the beverage with 2% hemp oil had the lowest amount of the bacteria in question.

The initial number of *Lactobacillus delbrueckii* ssp. *bulgaricus* was lower than that of *S. thermophilus* and ranged from 8.8 to 8.1 log CFU/mL in the fresh fermented milk drinks. After 28 days of refrigerated storage, *Lactobacillus* viability did not decrease significantly and ranged from 8.6 to 8.2 log CFU/mL. The initial and final amounts of viable Lactobacillus bacteria were the same in the control sample and with 2% hemp extract added. The most significant variation in the number of bacteria was observed in the drinks sample containing 2% hemp oil. Based on the statistical analysis, it was found that the addition of the hemp batch alone did not significantly inhibit the growth of both *S. thermophilus* and *Lactobacillus* bacteria. However, the final number of the bacteria in question in the product after fermentation and after 28 days of storage is influenced by the amount of hemp additive used (e.g., 2% hemp oil).

The main determinant of fermented dairy beverages’ potential therapeutic and prophylactic properties is the presence and number of yoghurt and probiotic bacteria cells throughout the declared shelf life at the recommended level (7 log CFU/mL) [1]. This requirement was met in all prepared samples throughout the shelf life. In addition, the values exceeded 8 log CFU/mL in all experimental samples, further enhancing the health-promoting potential of the finished product. According to EFSA (2010) claims, a bacterial content at such a high level improves lactose digestion in people with reduced intestinal lactase synthesis capacity [4,20,21].

Up to now, there have been no studies on the use of *Cannabis sativa* L. *var. sativa*-based additives (ethanolic hemp extract, hemp oil or dried hemp) in the production of fermented milk drinks. There is also a lack of information on the effect of these additives on bacterial survival. Research to date has looked at the addition of herbs or spices to yoghurt. Illupapalayam et al. [22] compared the effects of adding cinnamon, cardamom and nutmeg on bacterial survival during 28 days of refrigerated storage (4 °C). Depending on the strain used and the observed variability in the viability of these bacteria after 4 weeks of storage, all samples analysed contained acceptable levels of lactic fermentation bacteria. The results obtained by us and by Illupapalayam et al. [22] are comparable to the number of log colony-forming units in natural probiotic yoghurt and were 8 log CFU/mL or higher [22,23].

This is the first study demonstrating the potential use of the *Cannabis sativa* L. *var. sativa* plant in the food industry. However, it is important to see how the hemp batches used and the amounts of additives affect the survival of other important probiotic bacteria.

### 2.3. pH

Changes in pH before and after fermentation and during 28 days of storage at 4 °C are shown in Table 3.

During the fermentation process, the changes in pH decreased more in control samples containing ethanolic hemp extract (0.5%) and dried hemp (0.5 and 1%). This indicates differences in starter culture activity in the presence of the hemp inputs used. The initial pH of the milk was 6.6, and the addition of the different types of feedstock alone increased the pH of the milk with the hemp feedstock. The fermentation process was carried out until the pH was close to 4.6. The pH value chosen is optimal for adequate growth of both *Lactobacillus delbruecki* ssp. *bulgaricus* and *Streptococcus thermophilus* [24]. The length of fermentation depended on the amount and type of feedstock used. The fermentation process was shorter (3 h) for the control samples and those containing 0.5% hemp as extract and 0.5% and 1% dried hemp compared to the others, where it took 4 h. After the fermented milk beverage was produced, the pH of all samples ranged from 4.55 to 4.65, while after 24 h, the pH decreased and ranged from 4.40 to 4.55. During 28 days of refrigerated storage, a decrease in pH was found in all samples analysed. The control sample and the sample containing 0.5% hemp extract had the lowest pH (4.13) after storage. In contrast, the sample containing 2% dried hemp was found to have the highest pH value. It was found that the addition of hemp oil (in any quantity) and a higher proportion (1 and 2%) of the other feedstocks in the fermented milk drinks significantly prolonged the fermentation process.

This is the first study to analyse pH changes during fermentation and storage of fermented dairy drinks containing a hemp-based input. Samples containing dried hemp are most easily compared to those containing, for example, dried fruit pomace or herbs. Znamirowska et al. [24] prepared samples containing powdered dried apple pomace at 1.5% and 3%. The starter culture used by the authors was the same as in our work (YC-X11). The authors showed no pH difference between the control and dried apple pomace samples. Ziarno et al. [25] compared the effect of the amount of addition (0.2–5 wt%) of extract of selected herbs on fermentation efficiency. The herbs used were valerian (*Valeriana officinalis* L.), sage (*Salvia officinalis* L.), chamomile (*Matricaria chamomilla* L.), chaste (*Cistus* L.), lime blossom (*Tilia* L.), plantain (*Plantago lanceolata* L.) and valerian (*Althaea* L.). The observed pH changes during fermentation confirmed that the extracts used did not inhibit the activity of the lactic fermentation bacteria. It was found that a higher addition of herbal extracts resulted in a fermented beverage with a higher pH. The results obtained in the cited work are similar to those obtained in our work, suggesting that a hemp input is possible but requires more detailed research.

### 2.4. Sensory Quality of Fermented Milk Drinks with Hemp Inputs

**Figure 5:**
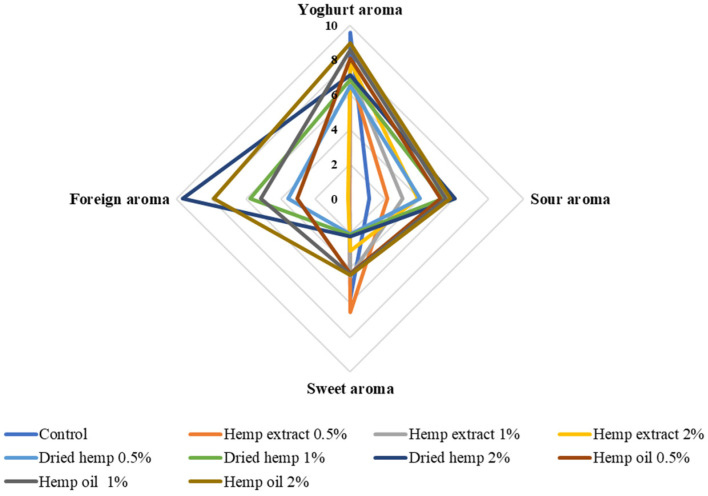
Aroma determinants of control fermented milk beverages and with hemp inputs (scale: 0—undetectable, 10—very intense).

**Figure 6:**
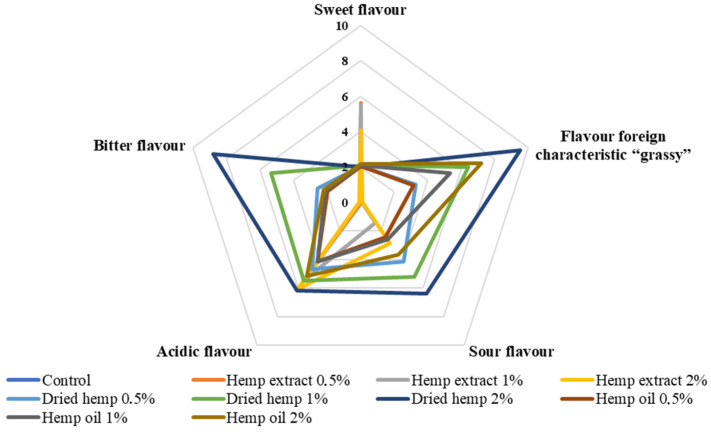
Flavour determinants of control fermented milk beverages and with hemp inputs (scale: 0—undetectable, 10—very intense).

**Figure 7:**
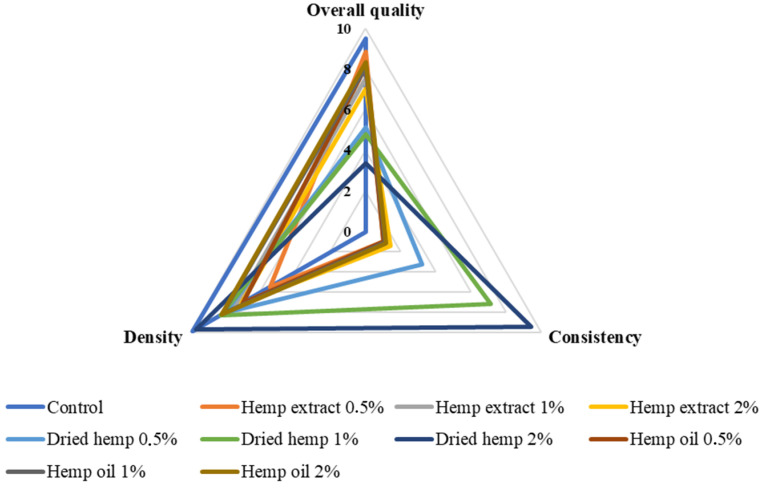
Density, consistency and overall quality of prepared fermented milk beverage control and with hemp inputs (scale 0–10; density: very thin—very thick; consistency: smooth—perceptible elements; overall quality: bad—very good).

The characteristics of the sensory quality attributes and the results of the QDA sensory evaluation of fresh fermented dairy beverages are shown in Figure 5, Figure 6 and Figure 7.

Among the attributes assessed were green colour and typical yoghurt colour, yoghurt aroma, sour, sweet, foreign ‘grassy’ aroma, sour taste, sour taste, bitter taste, consistency, density and overall quality of the prepared fermented dairy drinks. The prepared samples’ overall quality was negatively affected by the addition of dried hemp (all variants). The best overall quality (9.50) was obtained for the control sample due to it having the most suitable consistency density, the most noticeable aroma of fermented milk and the colour typical of traditional yoghurt. Of the fermented beverages containing hemp inputs, the best overall quality was characterised by samples containing 0.5% hemp extract and slightly lower scores were obtained for samples with the addition of hemp oil (all variants). The lowest overall quality was characterised by samples with 2% dried hemp added. This variant was rated lowest because it was characterised by a green colour, the least sweet smell, a significant extraneous ‘grassy’ taste and a bitter taste.

On the other hand, it should be mentioned that the perceived bitter taste and the inadequate texture containing lumps were due to the use of the dried product, which may have affected the sensory perception in the mouth of the panellists. The fermented dairy drinks containing the hemp oil inputs were rated very good in terms of texture, which was smooth and creamy in the mouth. Increasing the amount of fat in the samples resulted in improved viscosity and texture, which was positively received by the sensory panel. The samples with hemp extract added (all variants) had the colour most similar to typical yoghurt. These samples had the least acidic and extraneous ‘grassy’ smell. The sample variant containing 0.5% hemp extract was the least acidic of all samples containing hemp inputs.

This is the first study on the sensory analysis of fermented dairy drinks with hemp inputs in different forms and contents. Previous studies have focused on preparing samples with extracts of herbs or plants more common than *Cannabis sativa* L. and include cinnamon, cardamom, garlic and lemongrass, among others [22,26]. Illupapalayam et al. [22] conducted a sensory evaluation of yoghurts with spice oleoresins extracted from cardamom, cinnamon and nutmeg. The prepared yoghurts achieved a sensory acceptance higher than 7 IU (conventional units) in all cases. In our study, only yoghurts containing hemp extracts (each variant of additive quantity) were rated lower than 7 IU.

## 3. Materials and Methods

### 3.1. Materials

Freeze-dried hemp was prepared by freeze-drying the inflorescences of the *Cannabis sativa* L. *var. sativa* plant of the ‘Białobrzeskie’ variety. The plants were obtained from the Institute of Natural Fibers and Herbaceous Plants (Poznań-Pętkowo, Poland). The plants were harvested at the peak of flowering (between the twentieth day after the start of flowering and the tenth day after the end of flowering) [27]. Ethanolic hemp extract was obtained by cannabinoid extraction with ethanol using the method of Kanabus et al. [15]. Commercially available hemp oil (18% CBD + CBDA) as a dietary supplement was purchased from Cosma S.A. (Warsaw, Poland). Commercial milk (3.8% fat) was purchased from SM Mlekpol (Grajewo, Poland). Yoghurt culture YC-X11 Yo-Flex containing *Lactobacillus delbrueckii* ssp. *bularicus* and *Streptococsspcus thermophilus* was obtained from Chr. Hansen (Cząstków Mazowiecki, Poland).

### 3.2. Preparation of Fermented Milk Drinks

A control sample of fermented milk drinks without hemp was prepared, as well as experimental samples with 0.5, 1.0 and 2.0% (*w*/*v*) hemp inputs (hemp extract after extraction with ethanol, dried hemp and hemp oil). Due to the intense odour, colour and differences in consistency of the selected batches, it was decided to add smaller amounts to these batches in order to be able to develop this topic in the future. The hemp additives were weighed in test tubes, then added and mixed into milk. The samples were pasteurised at 93 ± 2 °C for 5 min, cooled to 42 ± 2 °C and inoculated by the addition of yoghurt starter culture YC-X11 at a concentration of 0.1 g/L of milk. After mixing thoroughly, the inoculated milk was incubated at 42 ± 2 °C until the pH reached 4.60 ± 0.05. The fermentation was stopped at this pH by cooling the products in an ice-water bath. Afterwards, set yoghurts were placed in the fridge at 4 °C for 28 days. Each batch of yoghurt was prepared in triplicate. The storage time and conditions adopted for the model products were intended to simulate the time (21–28 days on average) and conditions of shelf storage (4–6 °C). This study was designed to test the survival rate of lactic fermentation bacteria in fermented dairy beverages with the addition of different hemp inputs. Also, the aim was to check whether the different forms of the additives affect the quality and safety of the prepared product.

### 3.3. Chemicals and Reagents

The certified reference materials of cannabidiol (CBD), cannabidiolic acid (CBDA), cannabigerol (CBG), cannabichromene (CBC), cannabinol (CBN), cannabidiolic acid (CBNA), cannabidivarinic acid (CBDVA), cannabicyclol (CBL) and cannabicyclic acid (CBLA) were provided in 1.0 mg/mL solutions in methanol (MeOH) or acetonitrile (ACN) by Restek GmbH (Bad Homburg, Germany). Cannabigerolic acid (CBGA), cannabichromenic acid (CBCA), Δ^9^-tetrahydrocannabinol (Δ^9^-THC), Δ^8^-tetrahydrocannabinol (Δ^8^-THC), Δ^9^-tetrahydrocannabinolic acid A (Δ^9^-THCA-A), Δ^9^-tetrahydrocannabivarinic acid (Δ^9^-THCVA) and cannabidivarin (CBDV) were purchased from LGC Standards (Teddington, UK). Δ^9^-tetrahydrocannabivarin (Δ^9^-THCV) was provided in 1.0 mg/mL solutions in MeOH or ACN by Cerilliant Corporation (Round Rock, TX, USA). The certified purity value for all the CRMs was > 98.00%. Liquid chromatography–mass spectrometry (LC-MS)-grade water, ACN and MeOH were purchased from Witko (Łódź, Poland). Formic acid and ammonium formate (LC-MS grade) were obtained from Sigma Aldrich (St. Louis, MO, USA).

### 3.4. Preparation of Standard Solutions and Calibration Curves

Standard 100 μg/mL solutions for all 17 cannabinoids were prepared by dissolving 1.0 mL of the compound reference standard in ACN or MeOH using 10 mL volumetric flasks separately. This step was repeated as it was necessary to prepare higher dilutions for most compounds except CBD and CBDA. All solutions were stored at <−80 °C. Eight-point curves were prepared for 17 cannabinoids in different ranges, which were generated using Thermo TraceFinderTM software, version 5.1 (Thermo Fisher Scientific, Pleasanton, CA, USA).

### 3.5. Extraction of Cannabinoids from Fermented Milk Samples

The samples were mixed thoroughly before analysis. For each sample, 1 g of fermented milk was weighed in a 50 mL Falcon vial and extracted with 9 mL ACN. The samples were shaken for 15 min and then centrifuged (2 min 10,000 rpm) using an MPW-380R centrifuge from MPW Med. Instruments (Warsaw, Poland). For analysis, 1 mL of extract was filtered through a 0.22 µm 13 mm syringe filter (LLG Labware, Meckenheim, Germany).

### 3.6. Analysis of Cannabinoids by UHPLC-HESI-MS

Cannabinoids were analysed using an ultra-high-performance liquid chromatography-Q-Exactive Orbitrap mass spectrometry setup operating with a heated electrospray interface (UHPLC-HESI-MS) (Thermo Fisher Scientific, Waltham, MA, USA). Detailed information on the determination of cannabinoids is described by Kanabus et al. [15].

### 3.7. Method Validation

The results obtained for the development and validation of the cannabinoid determination method used in this article are shown in Table S1. To confirm and maintain the validity of the method used, control yoghurt samples were fortified at three levels for each cannabinoid. The recoveries achieved were within the target range of 80–120% and fulfilled the guidelines in ICH 2005 (https://www.ema.europa.eu/en/ich-q2r2-validation-analytical-procedures-scientific-guideline, accessed on 1 July 2024) and AOAC 2002 (https://aoac.org, accessed on 1 July 2024) [15].

### 3.8. Microbiological Analyses

The enumeration of yoghurt characteristic microorganisms was conducted after 1,7,14,21 and 28 days of storage at 4 °C in duplicates using plate techniques as reported in the ISO Standard (ISO 7889:2003) [28]. For S. thermophilus enumeration, M17 agar (Merck, Warsaw, Poland) was used, and the incubation was done aerobically at 37 °C for 48 h. Lactobacillus was enumerated using MRS agar (Merck) at pH 5.2 by anaerobic incubation (Biomeriux, Marcy-l’Étoile, France, GENbag anaer) at 37 °C for 72 h.

### 3.9. pH

The changes in pH of yoghurts were measured during fermentation and after 1, 7, 14, 21 and 28 days of storage at 4 °C in triplicate using a SevenExcellence pH meter S400 (Mettler-Toledo, Warsaw, Poland).

### 3.10. Sensory Analysis

Quantitative descriptive analysis (QDA) was carried out according to an ISO procedure (ISO 13299:2016) to evaluate the sensory properties of fermented dairy drink samples the day after manufacture [29]. QDA is a method recognised as a good tool for measuring the sensory attributes of different dairy products [30,31]. Sensory testing was carried out after obtaining approval from the University Ethical Committee (UEC) for Research with Human Participation Resolution No. UEC/14/2023 (UEC University of Life Sciences in Lublin). Sensory evaluation was carried out by a 6-member trained sensory panel. Approximately 5 g of each prepared fermented dairy drink variant was submitted for assessment. The panel selected and defined the attributes that most consistently described the yoghurt products. The following attributes were selected: colour green, typical of yoghurt, yoghurt aroma, sour, sweet, extraneous ‘grassy’, taste sweet, sour, sour, bitter, extraneous ‘grassy’, texture and density. The intensity of these attributes was measured using a 10-centimetre linear unstructured graphical scale, anchored from 0 (none) to 10 (very intense). In addition, based on the aforementioned distinguishing attributes, the overall sensory quality of the samples was further determined using a separate scale from 0 (low) to 10 (high). The results were expressed in IU (conventional units).

### 3.11. Statistical Analysis

All experiments and analyses performed were carried out in triplicate. The results are presented as mean measurement values. Statistical analyses were carried out using Statistica 13 software. The effects of hemp feed addition and storage time on the pH value, viable bacteria count and sensory evaluation of the prepared yoghurts were analysed by one-way ANOVA. Differences between the results were tested for significance (*p* < 0.05). The homogeneity of the groups was determined using the Tukey HSD test.

## 4. Conclusions

This study showed that the choice of hemp input form for producing a fermented dairy beverage influences the amount of cannabinoids in the final product. The type of hemp input also influences the sensory acceptance of the resulting fermented products. Samples containing hemp oil had the highest overall quality among the analysed variants. It was also shown that the cold storage time of the fermented dairy beverages did not significantly affect the reduction of lactic fermentation bacteria. Also, no pH value increase suggesting product spoilage was observed. Based on the results, the possibility of using hemp-based batches based on *Cannabis sativa* L. *var. sativa* for the production of fermented milk drinks was confirmed.

## Figures and Tables

**Table 1:** Total Δ^9^-THC content before and after fermentation and after 28 days of storage in fermented milk drinks with hemp inputs.

		Total Δ^9^-THC Content ^1^ [mg/100 g of Ready Product]		
	Amount of Hemp Additive % (*w*/*v*)	Before Fermentation	After Fermentation	After 4 Weeks of Storage
Sample with hemp oil	0.5	0.21 ^b^ ± 0.01	0.04 ^a^ ± 0.01	0.04 ^a^ ± 0.01
	1.0	0.42 ^b^ ± 0.04	0.05 ^a^ ± 0.01	0.05 ^a^ ± 0.01
	2.0	0.83 ^b^ ± 0.08	0.07 ^a^ ± 0.01	0.07 ^a^ ± 0.01
Sample with dried hemp	0.5	0.98 ^b^ ± 0.04	0.03 ^a^ ± 0.01	0.03 ^a^ ± 0.01
	1.0	1.98 ^b^ ± 0.09	0.05 ^a^ ± 0.01	0.05 ^a^ ± 0.01
	2.0	3.94 ^b^ ± 0.12	0.07 ^a^ ± 0.01	0.07 ^a^ ± 0.01
Sample with hemp extract	0.5	0.84 ^b^ ± 0.02	0.02 ^a^ ± 0.01	0.02 ^a^ ± 0.01
	1.0	1.68 ^b^ ±0.03	0.03 ^a^ ± 0.01	0.03 ^a^ ± 0.01
	2.0	3.34 ^b^ ± 0.06	0.06 ^a^ ± 0.01	0.06 ^a^ ± 0.01

^1^ The amount of Δ^9^-THC + (0.877 × amount of Δ^9^-THCA-A) [19]. ^a,b^—the different small letters within the same row indicate a significant difference (α < 0.05).

**Table 2:** Changes in microbial counts of yoghurt cultures (log cfu/mL) after fermentation and after 1, 7, 14, 21 and 28 days of storage at 4 °C in hemp-enriched fermented milk drinks.

	Amount of Hemp Additive	After Fermentation	Storage Time (Days)				
			1	7	14	21	28
*Streptococcus thermophilus*	Control	9.2 ^Ab^ ± 0.1	10.5 ^Bb^ ± 0.1	9.1 ^Aab^ ± 0.1	9.0 ^Aa^ ± 0.2	9.3 ^Aa^ ± 0.1	9.3 ^Aa^ ± 0.1
	Hemp extract 0.5%	8.7 ^Ac^ ± 0.1	9.0 ^Aba^ ± 0.3	9.1 ^Bab^ ± 0.1	9.0 ^Aba^ ± 0.2	9.1 ^Ba^ ± 0.2	9.1 ^Ba^ ± 0.1
	Hemp extract 1%	10.0 ^Cd^ ± 0.1	9.0 ^Aba^ ± 0.1	9.2 ^Bab^ ± 0.2	8.9 ^Aa^ ± 0.1	8.8 ^Aa^ ± 0.2	8.8 ^Aa^ ± 0.2
	Hemp extract 2%	8.9 ^Aac^ ± 0.1	9.2 ^Ba^ ± 0.1	9.0 ^Aa^ ± 0.1	8.9 ^Aa^ ± 0.1	9.0 ^Aba^ ± 0.1	9.0 ^Aba^ ± 0.1
	Dried hemp 0.5%	9.0 ^Aab^ ± 0.1	9.2 ^Aa^ ± 0.1	8.9 ^Aa^ ± 0.1	9.2 ^Aab^ ± 0.1	9.0 ^Aa^ ± 0.2	9.0 ^Aa^ ± 0.1
	Dried hemp 1%	9.1 ^Aab^ ± 0.1	9.0 ^Aa^ ± 0.1	8.9 ^Aa^ ± 0.1	9.3 ^Aab^ ± 0.3	9.2 ^Aa^ ± 0.5	9.2 ^Aa^ ± 0.5
	Dried hemp 2%	9.1 ^Bab^ ± 0.1	9.0 ^Aba^ ± 0.1	8.8 ^Aa^ ± 0.1	9.4 ^Cb^ ± 0.1	9.0 ^Aba^ ± 0.1	9.0 ^Aba^ ± 0.1
	Hemp oil 0.5%	8.9 ^Aabc^ ± 0.1	9.1 ^Aa^ ± 0.1	8.9 ^Aa^ ± 0.1	9.1 ^Aab^ ± 0.1	9.2 ^Aa^ ± 0.4	9.2 ^Aa^ ± 0.2
	Hemp oil 1%	9.0 ^Aabc^ ± 0.1	9.1 ^Aa^ ± 0.1	8.8 ^Aa^ ± 0.2	9.1 ^Aab^ ± 0.1	8.8 ^Aa^ ± 0.3	8.8 ^Aa^ ± 03
	Hemp oil 2%	9.0 ^Aabc^ ± 0.1	9.2 ^Aba^ ± 0.1	9.7 ^Bb^ ± 0.6	9.0 ^Aa^ ± 0.1	8.1 ^Cb^ ± 0.2	8.1 ^Cb^ ± 0.1
*Lactobacillus* *delbrueckii* ssp. *bulgaricus*	Control	8.5 ^Aab^ ± 0.1	8.2 ^Ba^ ± 0.1	8.3 ^Bad^ ± 0.1	8.4 ^Aa^ ± 0.1	8.5 ^Abc^ ± 0.1	8.5 ^Abc^ ± 0.1
	Hemp extract 0.5%	8.6 ^Cab^ ± 0.1	8.3 ^Aba^ ± 0.1	8.5 ^BC^ ± 0.1	8.2 ^Aa^ ± 0.1	8.2 ^Aa^ ± 0.2	8.2 ^Aa^ ± 0.1
	Hemp extract 1%	8.4 ^Aa^ ± 0.2	8.2 ^Aa^ ± 0.1	8.3 ^Aabd^ ± 0.1	9.2 ^Bb^ ± 0.2	8.2 ^Aa^ ± 0.2	8.2 ^Aa^ ± 0.1
	Hemp extract 2%	8.2 ^Ac^ ± 0.1	8.3 ^Aab^ ± 0.1	8.2 ^Aa^ ± 0.1	8.4 ^Aa^ ± 0.1	8.2 ^Aa^ ± 0.1	8.2 ^Aa^ ± 0.1
	Dried hemp 0.5%	8.8 ^Ab^ ± 0.1	8.6 ^ACe^ ± 0.1	8.4 ^BCabc^ ± 0.1	8.3 ^Ba^ ± 0.1	8.6 ^Ac^ ± 0.2	8.6 ^Ac^ ± 0.1
	Dried hemp 1%	8.6 ^Aab^ ± 0.1	8.6 ^Abc^ ± 0.1	8.3 ^Aabc^ ± 0.1	8.3 ^Aa^ ± 0.3	8.3 ^Aab^ ± 0.1	8.3 ^Aab^ ± 0.1
	Dried hemp 2%	8.4 ^Aba^ ± 0.1	8.4 ^ABab^ ± 0.1	8.4 ^Abe^ ± 0.1	8.5 ^Ca^ ± 0.1	8.3 ^Aabc^ ± 0.2	8.3 ^Aabc^ ± 0.1
	Hemp oil 0.5%	8.5 ^Bab^ ± 0.1	8.3 ^Aa^ ± 0.1	8.3 ^Aabd^ ± 0.1	8.3 ^Aa^ ± 0.1	8.3 ^Aabc^ ± 0.1	8.3 ^Aabc^ ± 0.1
	Hemp oil 1%	8.5 ^Aba^ ± 0.1	8.3 ^Aba^ ± 0.1	8.4 ^ABabc^ ± 0.1	8.5 ^Ba^ ± 0.2	8.2 ^Aa^ ± 0.1	8.2 ^Aa^ ± 0.1
	Hemp oil 2%	8.1 ^Ac^ ± 0.1	8.6 ^ABCb^ ± 0.1	8.8 ^Ce^ ± 0.1	8.6 ^BCa^ ± 0.1	8.2 ^Aba^ ± 0.2	8.2 ^Aba^ ± 0.1

^a–d^—the different small letters within the same row indicate a significant difference (α < 0.05) influenced by storage time in one type of yoghurt; ^A–C^—the different capital letters within the same column indicate significant differences (α < 0.05) influenced by the type and amount of hemp input to the yoghurt, e.g., hemp extract.

**Table 3:** Changes in pH after fermentation and during storage (4 weeks) of a fermented dairy drink with hemp input.

			Changes During Fermentation (h)				Changes During Storage (Week)				
	Milk UHT 3.8%	Milk with Hemp Additive	1	2	3	4	24 (h)	1	2	3	4
Control	6.60 ^a^ ± 0.05	-	5.88 ^Cb^ ± 0.05	4.87 ^Dc^ ± 0.03	4.58 ^Dd^* ± 0.02		4.50 ^Abe^ ± 0.02	4.22 ^Df^ ± 0.01	4.19 ^Eg^ ± 0.01	4.13 ^Dh^ ± 0.01	4.13 ^Dh^ ± 0.01
Hemp extract 0.5%		6.66 ^Aba^ ± 0.03	5.92 ^Bb^ ± 0.04	4.95 ^BCc^ ± 0.02	4.65 ^Cd^* ± 0.01		4.53 ^Abe^ ± 0.04	4.23 ^Df^ ± 0.01	4.22 ^Df^ ± 0.01	4.13 ^Dg^ ± 0.02	4.13 ^CDg^ ± 0.05
Hemp extract 1%		6.68 ^Aba^ ± 0.03	6.00 ^Bb^ ± 0.02	5.00 ^Bc^ ± 0.04	4.71 ^Bd^ ± 0.02	4.63 ^ABe^* ± 0.02	4.46 ^ABf^ ± 0.04	4.27 ^Cg^ ± 0.02	4.25 ^CDg^ ± 0.02	4.15 ^CDh^ ± 0.01	4.15 ^CDh^ ± 0.02
Hemp extract 2%		6.68 ^Aba^ ± 0.03	6.12 ^Ab^ ± 0.02	5.19 ^Ac^ ± 0.01	4.81 ^Ad^ ± 0.04	4.65 ^Ae^* ± 0.01	4.52 ^Af^ ± 0.02	4.32 ^Bg^ ± 0.01	4.27 ^Ch^ ± 0.02	4.20 ^Ci^ ± 0.01	4.17 ^Cij^ ± 0.03
Dried hemp 0.5%		6.61 ^Aa^ ± 0.03	5.52 ^Eb^ ± 0.04	4.75 ^Ec^ ± 0.04	4.55 ^Dd^* ± 0.03		4.40 ^Be^ ± 0.02	4.32 ^Bf^ ± 0.01	4.22 ^Dg^ ± 0.02	4.16 ^Ch^ ± 0.02	4.14 ^Di^ ± 0.04
Dried hemp 1%		6.64 ^Aa^ ± 0.01	5.52 ^Eb^ ± 0.04	4.77 ^Ec^ ± 0.05	4.57 ^Dd^* ± 0.01		4.46 ^Abe^ ± 0.04	4.35 ^Bf^ ± 0.02	4.31 ^Bf^ ± 0.03	4.26 ^Bg^ ± 0.03	4.18 ^Ch^ ± 0.03
Dried hemp 2%		6.65 ^Aa^ ± 0.04	5.63 ^Db^ ± 0.02	4.96 ^Cc^ ± 0.01	4.75 ^Bd^ ± 0.03	4.65 ^Ae^* ± 0.01	4.52 ^Af^ ± 0.02	4.55 ^Af^ ± 0.02	4.48 ^Ag^ ± 0.03	4.40 ^Ah^ ± 0.02	4.36 ^Ahi^ ± 0.03
Hemp oil 0.5%		6.60 ^Aa^ ± 0.02	5.89 ^Cb^ ± 0.05	4.94 ^BCc^ ± 0.05	4.71 ^BCd^ ± 0.05	4.59 ^Be^* ± 0.02	4.53 ^ABf^ ± 0.04	4.27 ^Cg^ ± 0.03	4.25 ^Cg^ ± 0.01	4.17 ^Ch^ ± 0.01	4.17 ^BCi^ ± 0.04
Hemp oil 1%		6.61 ^Aa^ ± 0.02	5.89 ^Cb^ ± 0.02	4.92 ^Cc^ ± 0.04	4.72 ^Bd^ ± 0.05	4.58 ^Be^* ± 0.02	4.48 ^ABf^ ± 0.03	4.27 ^Cg^ ± 0.01	4.25 ^Cg^ ± 0.01	4.20 ^Ch^ ± 0.02	4.20 ^BCh^ ± 0.05
Hemp oil 2%		6.67 ^Aba^ ± 0.04	5.98 ^Bb^ ± 0.05	5.04 ^Bc^ ± 0.06	4.75 ^ABd^ ± 0.06	4.65 ^Ae^* ± 0.01	4.55 ^Af^ ± 0.04	4.27 ^Cg^ ± 0.01	4.26 ^Cg^ ± 0.02	4.25 ^Bg^ ± 0.02	4.24 ^Bgh^ ± 0.05

*—end of fermentation. Data are presented as mean ± standard deviation (n = 3). Means with different letters within rows (^a–j^) and within columns (^A–E^) differ significantly (α < 0.05).

## Data Availability

The original contributions presented in this study are included in the article/Supplementary Materials; further inquiries can be directed to the corresponding author.

## Supplementary Materials

The following supporting information can be downloaded at: https://www.mdpi.com/article/10.3390/molecules29215056/s1, Table S1: Correlation coefficient, analytical ranges, limit of detection (LOD), and limit of quantification (LOQ); Table S2: Recovery rate (R%) and method repeatability (expressed as relative standard deviation, RSD%) for individual analytes at three different fortification levels (n = 5); Table S3: Cannabinoids determined in fermented milk drink with hemp extract input (0.5%, 1% and 2% (*w/v*)); Table S4: Cannabinoids determined in fermented milk drink with dried hemp input (0.5%, 1% and 2% (*w*/*v*)); Table S5: Cannabinoids determined in fermented milk drink with dried hemp input (0.5%, 1% and 2% (*w*/*v*)); Table S6: Sensory quality determinants used in the profile evaluation of samples with hemp input; Table S7: Results of the profile analysis of hemp-infused fermented milk drinks (0–10 scale).

## References

[1] FAO/WHO Food Standard Codex Alimentarius Commission. 2003. Codex Standard for Fermented Milks: Codex STAN 243. https://www.fao.org/fao-who-codexalimentarius/sh-proxy/en/?lnk=1&url=https%253A%252F%252Fworkspace.fao.org%252Fsites%252Fcodex%252FStandards%252FCXS%2B243-2003%252FCXS_243e.pdf.

[2] Hill B.C., Guarner F., Reid G., Gibson G.R., Merenstein D.J., Pot B., Morelli L., Canani B.C., Flint H.J., Salminen S. (2014). Expert consensus document: The International Scientific Association for Probiotics and Prebiotics consensus statement on the scope and appropriate use of the term probiotic. Nat. Rev. Gastroenterol. Hepatol..

[3] Lee W.J., Lucey J.A. (2010). Formation and Physical Properties of Yoghurt. Asian-Australasian J. Anim. Sci..

[4] Kycia K., Chlebowska-Śmigiel A., Szydłowska A., Sokół E., Ziarno M., Gniewosz M. (2020). Pullulan as a potential enhancer of Lactobacillus and Bifidobacterium viability in symbiotic low fat yoghurt and its sensory quality. LWT Food Sci. Technol..

[5] Zare F., Boye J., Orst V., Champagne C., Simpson B. (2011). Microbial, physical and sensory properties of yoghurt supplemented with lentil flour. Food Res. Int..

[6] Świderski F., Waszkiewicz-Robak B. (2010). Processed Food Commodities with Elements of Technology.

[7] Cisło K., Szostak K., Wołanciuk A., Kędzierska-Matysek M., Dopieralska P. (2018). Characteristics of fermented milk drinks using yoghurt as an example. Bioeconomy and the Environment.

[8] Świderski F., Kolanowski W. (1999). New food product containing polyunsaturated fatty acids omega-3 epa, dha—sensory quality and possibility of diet supplementation. Ann. Natl. Inst. Hyg..

[9] Kanabus J., Bryła M., Roszko M., Modrzewska M., Pirzegalski A. (2021). Cannabinoids—Characteristics and potential for use in food production. Molecules.

[10] Markowska J., Polak E., Drabent A., Żak A. (2021). *Cannabis sativa* L.—Varieties, properties. Food Sci. Technol. Qual..

[11] Granato D., Santos J.S., Salem R.D.S., Mortazavian A.M., Rocha R.S., Cruz A.G. (2018). Effects of herbal extracts and quality traits of yogurts, cheeses, fermented milks, and ice creams: A technological perspective. Curr. Opin. Food Sci..

[12] Citti C., Pacchetti B., Vandelli M.A., Forni F., Cannazza G. (2018). Analysis of cannabinoids in commercial hemp seed oil and decarboxylation kinetics studies of cannabidiolic acid (CBDA). J. Pharm. Biomed. Anal..

[13] Garrett E.R.C., Hunt A. (1974). Physicochemical properties, solubility, and protein binding of Δ9-tetrahydrocannabinol. J. Pharm. Sci..

[14] Gonçalves J., Rosado T., Soares S., Simão A.Y., Caramelo D., Luís Â., Fernández N., Barroso M., Gallardo E., Duarte A.P. (2019). Cannabis and its secondary metabolites: Their use as therapeutic drugs, toxicological aspects, and analytical determination. Medicines.

[15] Kanabus J., Bryła M., Roszko M. (2023). The Development, Validation, and Application of a UHPLC-HESI-MS Method for the Determination of 17 Cannabinoids in *Cannabis sativa* L. var. sativa Plant Material. Molecules.

[16] Garcia-Valverde M.T., Snachez-Carnerero Callado C., Diaz-Linan M.C., Sanchez de Medina V., Hidalgo-Garcia J., Nadal X., Hanus L., Ferreiro-Vera C. (2022). Effect of temperature in the degradation of cannabinoids: From a brief residence in the gas chromatography inlet port to a longer period in thermal treatments. Front. Chem..

[17] (2015). Scientific Opinion on the Risks for Human Health Related to the Presence of Tetrahydrocannabinol (THC) in Milk and Other Food of Animal Origin. EFSA J..

[18] (2020). EIHA Contribution on Maximum Levels for THC in Food. https://eiha.org/wp-content/uploads/2020/12/EIHA-contribution-on-THC-maximum-levels-in-food.pdf.

[19] Commission Regulation (EU) 2023/915 of 23 April 2023. https://eur-lex.europa.eu/eli/reg/2023/915/oj.

[20] (2010). Scientific Opinion on the Substantiation of Health Claims Related to Live Yoghurt Cultures and Improved Lactose Digestion (ID 1143, 2976) Pursuant to Article 13(1) of Regulation (EC) No 1924/2006. EFSA Panel Diet. Prod. Nutr. Allerg. (NDA).

[21] Łopusiewicz Ł., Waszkowiak K., Polanowska K., Mikołajczak B., Śmietana N., Hrebień-Flisińska A., Sadowska J., Mazurkiewicz-Zapałowicz K., Drozłowska E. (2022). The effect of yogurt and kefir starter cultures on bioactivity of fermented industrial by-product from Cannabis sativa production—hemp press cake. Fermentation.

[22] Illupapalayam V.V., Smith S.C., Gamlath S. (2014). Consumer acceptability and antioxidant potential of probiotic-yogurt with spices. LWT-Food Sci. Technol..

[23] Cruz A.G., Cadena R.S., Faria J.A.F., Bolini H.M.A., Dantas C., Ferreira M.M.C., Deliza R. (2012). PARAFAC: Adjustment for modelling consumer study covering probiotic and conventional yogurt. Food Res. Int..

[24] Znamirowska A., Kalicka D., Buniowska M., Rożek P. (2018). Effect of the addition of dried apple pomace on the physicochemical and sensory properties og yoghurts. Food. Science. Technology. Quality..

[25] Ziarno M., Kozłowska M., Ścibisz I., Kowalczyk M., Pawelec S., Stochmal A., Szleszyński B. (2021). The effect of selected herbal extracts on lactic acid bacteria activity. Appl. Sci..

[26] Helal A., Tagliazucchi D. (2018). Impact of in-vitro gastro-panceatic digestion on polyphenols and cinnamaldehyde bioaccessibility and antioxidant activity in stirred cinnamon-fortified yogurt. LWT-Food Sci. Technol..

[27] Commission Delegated Regulation (EU) 2017/1155 of 15 February 2017. https://eur-lex.europa.eu/eli/reg_del/2017/1155/oj.

[28] (2003). Yoghurt–Enumeration of Characteristic Microorganisms–Colony-Count Technique at 37 Degrees C.

[29] (2016). Sensory Analysis–Methodology–General Guidance for Stablishing a Sensory Profile.

[30] Silva H.L.A., Balthazar C.F., Silva R., Vieira A.H., Costa R.G.B., Esmerino E.A., Freitas M.Q., Cruz A.G. (2018). Sodium reduction and flavour enhancer addition in probiotic prato cheese: Contributions of quantitative descriptive analysis and temporal dominance of sensations for sensory profiling. J. Dairy Sci..

[31] Ramírez-Rivera E., Díaz-Rivera P., Ramón-Canul L.G., Juárez-Barrientos J.M., Rodríguez-Miranda J., Herman-Lara E., Prinyawiwatkul W., Herrera-Corredor J.A. (2018). Comparison of performance and quantitative descriptive analysis sensory profiling and its relationship to consumer liking between the artisanal cheese producers panel and the descriptive trained panel. J. Dairy Sci..

