# Supplementary material for: Exploring the Presence of Cannabinoids in Hemp-Infused Fermented Milk Drinks: An Analysis of Pre- and Post-Fermentation Levels
Source: Molecules. 2024 Oct 26;29(21):5056. doi: 10.3390/molecules29215056 (PMC11547984; doi:10.3390/molecules29215056)
Supplement: Supplementary file 1 [file molecules-29-05056-s001.zip › molecules-3256564-supplementary.pdf]

SUPPLEMENTARY MATERIALS

Exploring the Presence of Cannabinoids in Hemp-Infused Fermented Milk Drinks: An Analysis of Pre- and Post-Fermentation Levels

Joanna Kanabus*, Marcin Bryła, Katarzyna Kycia, Joanna Markowska, Marek Roszko

Table S1. Correlation coefficient , analytical ranges, limit of detection (LOD), and limit of quantification (LOQ)

**Analyte**	**Correlation coefficient (R^2)**	**Analytical ranges (µg/mL)**	**LOD (µg/mL)**	**LOQ (µg/mL)**
CBD	0.9993	0.2-25.6	0.001	0.003
CBDA	0.9999	0.4-51.2	0.0003	0.0009
Δ^9-THC	0.9999	0.08-10.24	0.0011	0.0036
Δ^9-THCA-A	0.9999	0.08-10.24	0.0004	0.0013
CBC	0.9997	0.02-2.56	0.005	0.02
CBCA	0.9992	0.08-10.24	0.0008	0.003
CBG	0.9994	0.008-1.024	0.0003	0.0009
CBGA	0.9992	0.02-2.56	0.00003	0.0001
CBDV	0.9998	0.008-1.024	0.001	0.002
CBDVA	0.9999	0.02-2.56	0.00003	0.0001
Δ^9-THCV	0.9999	0.008-1.024	0.0006	0.002
Δ^9-THCVA	0.9996	0.02-2.56	0.0001	0.0002
Δ^8-THC	0.9998	0.008-1.024	0.002	0.006
CBN	0.9998	0.008-1.024	0.002	0.007
CBNA	0.9996	0.002-0.256	0.0001	0.0002
CBL	0.9999	0.02-2.56	0.002	0.007
CBLA	0.9997	0.02-2.56	0.0004	0.0012

Table S2. Recovery rate (R%) and method repeatability (expressed as relative standard deviation, RSD%) for individual analytes at three different fortification levels (n=5).

Nominal concentration (μg/mL)	Measured concentration (μg/mL)	R (%)	RSD (%)
	CBD		
0.2	0.205	103	2
1.6	1.56	98	3
8.0	7.75	97	1
	CBDA		
1.0	1.02	102	2
2.0	2.11	105	2
4.0	3.98	100	2
	Δ^9-THC		
0.2	0.194	97	2
0.6	0.58	97	1
1.2	1.153	96	1
	Δ^9-THCA-A		
0.1	0.097	97	2
0.6	0.59	98	2
1.2	1.18	98	3
	CBC		
0.1	0.088	88	6
0.3	0.269	90	3
0.9	0.871	97	3
	CBCA		
0.1	0.102	102	2
1.0	0.948	95	2
2.0	1.982	99	1
	CBG		
0.005	0.0048	96	6
0.01	0.098	98	3
0.02	0.019	97	8
	CBGA		
0.1	0.1	100	3
0.3	0.3	100	1
0.6	0.57	95	5
	CBDV		
0.1	0.097	97	1
0.3	0.296	99	2
0.6	0.592	99	1
	CBDVA		
0.1	0.099	99	2
0.3	0.301	100	1
0.6	0.595	99	1
	Δ^9-THCV		
0.05	0.049	99	3
0.1	0.100	100	3
0.2	0.197	98	3
	Δ^9-THCVA		

0.05	0.049	98	2
0.1	0.098	98	3
0.2	0.195	97	2
		Δ^8-THC	
0.05	0.048	96	6
0.1	0.10	100	6
0.2	0.200	100	2
		CBN	
0.1	0.098	98	1
0.3	0.299	100	2
0.6	0.608	101	2
		CBNA	
0.005	0.005	100	2
0.01	0.0099	99	2
0.02	0.019	98	3
		CBL	
0.05	0.049	99	9
0.1	0.098	98	1
0.2	0.19	97	2
		CBLA	
0.05	0.05	100	2
0.1	0.099	99	2
0.2	0.198	99	1

Table S3. Cannabinoids determined in fermented milk drink with hemp extract input (0.5%, 1% and 2% (*w*/*v*)).

	Amount in fermented milk drink (mg/100g of product)																	
Analyte	Assumed amount of compound			After fermentation			1 week			2 weeks			3 weeks			4 weeks		
	0.5%	1%	2%	0.5%	1%	2%	0.5%	1%	2%	0.5%	1%	2%	0.5%	1%	2%	0.5%	1%	2%
CBD	0.25^{Af} ± 0.02	0.50^{Bd} ± 0.04	1.01^{Cc} ± 0.08	0.07^{Ade} ± 0.01	0.09^{ABb} ± 0.01	0.22^{Cb} ± 0.02	0.05^{Ad} ± 0.01	0.08^{Bb} ± 0.01	0.22^{Cb} ± 0.02	0.02^{Abc} ± 0.01	0.05^{Ba} ± 0.01	0.21^{Cb} ± 0.02	0.01^{Ab} ± 0.01	0.09^{Bb} ± 0.01	0.21^{Cb} ± 0.02	0.007^{Aa} ± 0.001	0.014^{Bc} ± 0.002	0.021^{Ca} ± 0.001
CBDA	1.87^{Ad} ± 0.13	3.75^{Bd} ± 0.26	7.4^{9Cf} ± 0.50	0.08^{Aab} ± 0.01	0.15^{Bc} ± 0.01	0.25^{Ce} ± 0.01	0.07^{Aa} ± 0.01	0.12^{Bab} ± 0.01	0.22^{Cc} ± 0.01	0.09^{Abc} ± 0.01	0.16^{Bc} ± 0.01	0.25^{Cd} ± 0.01	0.08^{Aab} ± 0.01	0.11^{Ba} ± 0.01	0,19^{Cab} ± 0.01	0.08^{Aab} ± 0.01	0.10^{ABa} ± 0.01	0.17^{Ca} ± 0.01
Δ^{9}-THC	0.42^{Ac} ± 0.03	0.84^{Bb} ± 0.06	1.67^{Cb} ± 0.11	0.01^{Ab} ± 0.01	0.01^{Aa} ± 0.01	0.02^{ABa} ± 0.01	0.01^{Ab} ± 0.01	0.01^{Aa} ± 0.01	0.02^{AB} ± 0.01	0.01^{Ab} ± 0.01	0.01^{Aa} ± 0.01	0.02^{ABaa} ± 0.01	0.004^{Aa} ± 0.001	0.01^{BCa} ± 0.01	0.02^{Ca} ± 0.01	0.004^{Aa} ± 0.001	0.01^{BCa} ± 0.01	0.02^{Ca} ± 0.01
Δ^{9}-THCA-A	0.48^{Ab} ± 0.03	0.96^{Bb} ± 0.07	1.91^{Cb} ± 0.03	0.01^{Aa} ± 0.01	0.02^{ABa} ± 0.01	0.04^{Ca} ± 0.01	0.01^{Aa} ± 0.01	0.02^{ABa} ± 0.01	0.03^{Ba} ± 0.01	0.01^{Aa} ± 0.01	0.02^{ABa} ± 0.01	0.03^{Ba} ± 0.01	0.01^{Aa} ± 0.01	0.02^{ABa} ± 0.01	0.03^{Ba} ± 0.01	0.01^{Aa} ± 0.01	0.02^{ABa} ± 0.01	0.03^{Ba} ± 0.01
CBC	0.04^{Ab} ± 0.01	0.08^{Bb} ± 0.01	0.17^{Cb} ± 0.07	ND^{Aa}	ND^{Aa}	ND^{Aa}	ND^{Aa}	ND^{Aa}	ND^{Aa}	ND^{Aa}	ND^{Aa}	ND^{Aa}	ND^{Aa}	ND^{Aa}	ND^{Aa}	ND^{Aa}	ND^{Aa}	ND^{Aa}
CBCA	0.54^{Ac} ± 0.04	1.08^{Bb} ± 0.08	2.16^{Cc} ± 0.16	0.01^{Ab} ± 0.01	0.02^{ABa} ± 0.01	0.04^{Cab} ± 0.01	ND^{Aa}	0.02^{Ba} ± 0.01	0.04^{Cab} ± 0.01	ND^{Aa}	0.02^{Ba} ± 0.01	0.04^{Cab} ± 0.01	ND^{Aa}	0.02^{Ba} ± 0.01	0.03B^{Ca} ± 0.01	ND^{Aa}	0.02^{Ba} ± 0.01	0.03^{BCa} ± 0.01
CBG	0.31^{Ab} ± 0.02	0.62^{Bb} ± 0.04	1.23^{Cb} ± 0.08	ND^{Aa}	ND^{Aa}	ND^{Aa}	ND^{Aa}	ND^{Aa}	ND^{Aa}	ND^{Aa}	ND^{Aa}	ND^{Aa}	ND^{Aa}	ND^{Aa}	ND^{Aa}	ND^{Aa}	ND^{Aa}	ND^{Aa}
CBGA	0.07^{Ab} ± 0.01	0.14^{Bc} ± 0.01	0.29^{Cb} ± 0.02	0.002^{Aa} ± 0.001	0.004^{ABb} ± 0.001	0.006^{BCa} ± 0.001	0.002^{Aa} ± 0.001	0.004^{ABa} ± 0.001	0.006^{BCa} ± 0.001	0.002^{Aa} ± 0.001	0.004^{ABa} ± 0.001	0.006^{BCa} ± 0.001	0.002^{Aa} ± 0.001	0.004^{ABa} ± 0.001	0.006^{BCa} ± 0.001	0.002^{Aa} ± 0.001	0.004^{ABa} ± 0.001	0.006^{BCa} ± 0.001
CBDV	0.003^{Ab}± 0.001	0.005^{ABb} ± 0.001	0.01^{Cb} ± 0.01	ND^{Aa}	ND^{Aa}	ND^{Aa}	ND^{Aa}	ND^{Aa}	ND^{Aa}	ND^{Aa}	ND^{Aa}	ND^{Aa}	ND^{Aa}	ND^{Aa}	ND^{Aa}	ND^{Aa}	ND^{Aa}	ND^{Aa}
CBDVA	0.04^{Ab} ± 0.01	0.08^{Bb} ± 0.01	0.16^{Cb} ± 0.01	0.001^{Aa} ± 0.001	0.003^{ABa} ± 0.001	0.004^{BCa} ± 0.001	0.001^{Aa} ± 0.001	0.003^{ABa} ± 0.001	0.004^{BCa} ± 0.001	0.001^{Aa} ± 0.001	0.003^{ABa} ± 0.001	0.004^{BCa} ± 0.001	0.001^{Aa} ± 0.001	0.003^{ABa} ± 0.001	0.004^{BCa} ± 0.001	0.001^{Aa} ± 0.001	0.003^{ABa} ± 0.001	0.004^{BCa} ± 0.001
Δ^{9}-THCV	0.003^{Ab} ± 0.001	0.005^{Bb} ± 0.001	0.01^{Cb} ± 0.01	ND^{Aa}	ND^{Aa}	ND^{Aa}	ND^{Aa}	ND^{Aa}	ND^{Aa}	ND^{Aa}	ND^{Aa}	ND^{Aa}	ND^{Aa}	ND^{Aa}	ND^{Aa}	ND^{Aa}	ND^{Aa}	ND^{Aa}
Δ^{9}-THCVA	0.02^{Ab} ± 0.01	0.03Ab ± 0.01	0.06Bb ± 0.01	ND^{Aa}	ND^{Aa}	ND^{Aa}	ND^{Aa}	ND^{Aa}	ND^{Aa}	ND^{Aa}	ND^{Aa}	ND^{Aa}	ND^{Aa}	ND^{Aa}	ND^{Aa}	ND^{Aa}	ND^{Aa}	ND^{Aa}
Δ^{8}-THC	ND^{Aa}	ND^{Aa}	ND^{Aa}	ND^{Aa}	ND^{Aa}	ND^{Aa}	ND^{Aa}	ND^{Aa}	ND^{Aa}	ND^{Aa}	ND^{Aa}	ND^{Aa}	ND^{Aa}	ND^{Aa}	ND^{Aa}	ND^{Aa}	ND^{Aa}	ND^{Aa}
CBN	ND^{Aa}	0.01^{Ab} ± 0.01	0.02^{Bb} ± 0.01	ND^{Aa}	ND^{Aa}	ND^{Aa}	ND^{Aa}	ND^{Aa}	ND^{Aa}	ND^{Aa}	ND^{Aa}	ND^{Aa}	ND^{Aa}	ND^{Aa}	ND^{Aa}	ND^{Aa}	ND^{Aa}	ND^{Aa}

CBNA	0.01^{Ab} ± 0.01	0.02^{ABb} ± 0.01	0.03^{Bb} ± 0.01	ND^{Aa}	ND^{Aa}	ND^{Aa}	ND^{Aa}	ND^{Aa}	ND^{Aa}	ND^{Aa}	ND^{Aa}	ND^{Aa}	ND^{Aa}	ND^{Aa}	ND^{Aa}	ND^{Aa}	ND^{Aa}	ND^{Aa}
CBL	ND^{Aa}	ND^{Aa}	ND^{Aa}	ND^{Aa}	ND^{Aa}	ND^{Aa}	ND^{Aa}	ND^{Aa}	ND^{Aa}	ND^{Aa}	ND^{Aa}	ND^{Aa}	ND^{Aa}	ND^{Aa}	ND^{Aa}	ND^{Aa}	ND^{Aa}	ND^{Aa}
CBLA	0.01^{Ab} ± 0.01	0.02^{ABb} ± 0.01	0.04^{Cb} ± 0.01	ND^{Aa}	ND^{Aa}	ND^{Aa}	ND^{Aa}	ND^{Aa}	ND^{Aa}	ND^{Aa}	ND^{Aa}	ND^{Aa}	ND^{Aa}	ND^{Aa}	ND^{Aa}	ND^{Aa}	ND^{Aa}	ND^{Aa}
Sum of 17 cannabinoids	4.07^{Ad} ± 0.16	8.14^{Bd} ± 0.33	16.26^{Cd} ± 0.65	0.18^{Ac} ± 0.01	0.30^{Bc} ± 0.01	0.58^{Cc} ± 0.02	0.14^{Ab} ± 0.01	0.26^{Bb} ± 0.01	0.54^{Cb} ± 0.02	0.13^{Ab} ± 0.01	0.27^{Bb} ± 0.01	0.56^{Cbc} ± 0.02	0.11^{Aab} ± 0.01	0.26^{Bb} ± 0.01	0.49^{Cb} ± 0.02	0.10^{Aa} ± 0.01	0.17^{Ba} ± 0.01	0.28^{Ca} ± 0.01

a-f – the different small letters within the same row (in one amount of the input used, for example, 0.5%) indicate a significant differences (α <0.05) influenced by storage time; A-C – the different capital letters within the same row indicate significant differences (α <0.05) influenced by the differences between the content of the analyzed substance in each week of storage

Table S4. Cannabinoids determined in fermented milk drink with dried hemp input (0.5%, 1% and 2% (*w/v*)).

	Amount in fermented milk drink (mg/100g of product)																	
**Analyte**	**Assumed amount of compound**			**After fermentation**			**1 week**			**2 weeks**			**3 weeks**			**4 weeks**		
	0.5%	1%	2%	0.5%	1%	2%	0.5%	1%	2%	0.5%	1%	2%	0.5%	1%	2%	0.5%	1%	2%
CBD	0.40^{Aa} ± 0.03	0.80^{Bab} ± 0.04	1.62^{Cc} ± 0.07	0.40^{Aa} ± 0.03	0.83^{Bb} ± 0.01	1.59^{Cb} ± 0.03	0.40^{Aa} ± 0.02	0.83^{Bb} ± 0.04	1.50^{Ca} ± 0.09	0.40^{Aa} ± 0.02	0.82^{Bb} ± 0.04	1.55^{Cab} ± 0.05	0.44^{Ab} ± 0.01	0.80^{Bab} ± 0.05	1.62^{Cc} ± 0.07	0.44^{Ab} ± 0.03	0.78^{Ba} ± 0.03	1.65^{Cd} ± 0.01
CBDA	2.66^{Aab} ± 0.19	5.32^{Bd} ± 0.37	10.64^{Cf} ± 0.75	2.86^{Ac} ± 0.05	5.38^{Bd} ± 0.12	9.50^{Ce} ± 0.05	2.80^{Abc} ± 0.11	5.21^{Bd} ± 0.14	9.25^{Cd} ± 0.10	2.71^{Ab} ± 0.13	$4,91^{Bbc}$ ± 0.11	9.00^{Cc} ± 0.15	2.64^{Aab} ± 0.11	4.87^{Bb} ± 0.12	8.50^{Cb} ± 0.15	2.55^{Aa} ± 0.06	4.68^{Ba} ± 0.12	8.14^{Ca} ± 0.17
Δ^9-THC	0.34^{Ab} ± 0.03	0.68^{Bb} ± 0.05	1.36^{Cb} ± 0.10	0.01^{Aa} ± 0.01	0.02^{Ba} ± 0.01	0.05^{Ca} ± 0.01	0.01^{Aa} ± 0.01	0.02^{Ba} ± 0.01	0.05^{Ca} ± 0.01	0.01^{Aa} ± 0.01	0.02^{Ba} ± 0.01	0.05^{Ca} ± 0.01	0.01^{Aa} ± 0.01	0.02^{Ba} ± 0.01	0.05^{Ca} ± 0.01	0.01^{Aa} ± 0.01	0.02^{Ba} ± 0.01	0.05^{Ca} ± 0.01
Δ^9-THCA-A	0.74^{Ab} ± 0.05	1.48^{Bbd} ± 0.10	2.95^{Cb} ± 0.20	0.02^{Aa} ± 0.01	0.03^{ABa} ± 0.02	0.02^{Aa} ± 0.02	0.02^{Aa} ± 0.01	0.03^{ABa} ± 0.02	0.02^{Aa} ± 0.02	0.02^{Aa} ± 0.01	0.03^{ABa} ± 0.02	0.02^{Aa} ± 0.02	0.02^{Aa} ± 0.01	0.03^{ABa} ± 0.02	0.02^{Aa} ± 0.02	0.02^{Aa} ± 0.01	0.03^{ABa} ± 0.02	0.02^{Aa} ± 0.02
CBC	0.07^{Acd} ± 0.01	0.13^{Bbc} ± 0.01	0.26^{Cd} ± 0.02	0.06^{Abc} ± 0.01	0.12^{Bab} ± 0.01	0.19^{Ca} ± 0.01	0.04^{Aa} ± 0.01	0.12^{Bab} ± 0.01	0.21^{Cab} ± 0.02	0.05^{Aab} ± 0.01	0.11^{Ba} ± 0.01	0.22^{Cb} ± 0.02	0.06^{Abc} ± 0.01	0.12^{Bab} ± 0.01	0.24^{Cc} ± 0.02	0.08^{Ad} ± 0.01	0.13^{Bbc} ± 0.01	0.26^{Cd} ± 0.01
CBCA	0.72^{Acd} ± 0.05	1.45^{Bd} ± 0.07	2.89^{Cb} ± 0.20	0.67^{Ac} ± 0.07	1.36^{Bc} ± 0.08	2.61^{Ca} ± 0.05	0.67^{Ac} ± 0.05	1.34^{Bc} ± 0.08	2.60^{Ca} ± 0.05	0.58^{Aa} ± 0.02	1.30^{Bbc} ± 0.08	2.63^{Ca} ± 0.04	0.60^{Aab} ± 0.07	1.26^{Bab} ± 0.07	2.60^{Ba} ± 0.04	0.60^{Aab} ± 0.02	1.24^{Ba} ± 0.02	2.63^{Ca} ± 0.01
CBG	0.38^{Ad} ± 0.03	0.76^{Be} ± 0.06	1.51^{Ce} ± 0.10	0.45^{Ae} ± 0.04	0.97^{Bf} ± 0.04	1.92^{Cf} ± 0.04	0.33^{Ac} ± 0.05	0.56^{Bd} ± 0.08	1.29^{Cd} ± 0.04	0.30^{Abc} ± 0.03	0.48^{Bc} ± 0.06	0.99^{Cc} ± 0.04	0.24^{Ab} ± 0.07	0.36^{Bb} ± 0.05	0.75^{Cb} ± 0.11	0.15^{Aa} ± 0.02	0.31^{Ba} ± 0.02	0.63^{Ca} ± 0.01
CBGA	0.10^{Ab} ± 0.01	0.20^{Bc} ± 0.02	0.40^{Ccd} ± 0.03	0.11^{Abc} ± 0.01	0.22^{Bd} ± 0.01	0.41^{Ccd} ± 0.01	0.10^{Ab} ± 0.01	0.18^{Bb} ± 0.02	0.39^{Cc} ± 0.04	0.09^{Aab} ± 0.01	0.16^{Ba} ± 0.02	0.35^{Cb} ± 0.02	0.08^{Aa} ± 0.01	0.16^{Ba} ± 0.03	0.32^{Ca} ± 0.02	0.08^{Aa} ± 0.01	0.16^{Ba} ± 0.01	0.32^{Ca} ± 0.01
CBDV	0.005^{Aab} ± 0.001	0.01^{Ba} ± 0.01	0.02^{Ca} ± 0.01	0.01^{ABcd} ± 0.01	0.02^{Bab} ± 0.01	0.03^{BCab} ± 0.01	0.01^{Acd} ± 0.01	0.01^{Aa} ± 0.01	0.02^{ABa} ± 0.01	0.009^{Ac} ± 0.001	0.01^{Aa} ± 0.01	0.02^{Ba} ± 0.01	0.005^{Ab} ± 0.001	0.01^{Ba} ± 0.01	0.02^{BCa} ± 0.01	0.004^{Aa} ± 0.001	0.01^{Ba} ± 0.01	0.02^{Ca} ± 0.01
CBDVA	0.05^{Aa} ± 0.01	0.09^{Ba} ± 0.01	0.18^{Ca} ± 0.01	0.06^{Aab} ± 0.01	0.12^{Bbc} ± 0.01	0.21^{Cb} ± 0.01	0.05^{Aa} ± 0.01	0.10^{Bab} ± 0.01	0.20^{Cab} ± 0.01	0.05^{Aa} ± 0.01	0.10^{Bab} ± 0.01	0.20^{Cab} ± 0.01	0.05^{Aa} ± 0.01	0.10^{Bab} ± 0.01	0.20^{Cab} ± 0.01	0.05^{Aa} ± 0.01	0.10^{Bab} ± 0.01	0.20^{Cab} ± 0.01
Δ^9-THCV	0.004^{Aab} ± 0.001	0.007^{Bab} ± 0.001	0.014^{Cc} ± 0.001	0.006^{Ac} ± 0.001	0.009^{Bbc} ± 0.001	0.016^{Cd} ± 0.001	0.004^{Aaba} ± 0.001	0.006^{ABa} ± 0.001	0.009^{Ca} ± 0.001	0.004^{Aab} ± 0.001	0.006^{ABa} ± 0.001	0.009^{Ca} ± 0.001	0.004^{Aab} ± 0.001	0.006^{ABa} ± 0.001	0.009^{Ca} ± 0.001	0.003^{Aa} ± 0.001	0.006^{Ba} ± 0.001	0.01^{Cab} ± 0.001
Δ^9-THCVA	0.02^{Aa} ± 0.01	0.05^{Ba} ± 0.01	0.10^{Cb} ± 0.01	0.02^{Aa} ± 0.01	0.05^{Ba} ± 0.01	0.09^{Ca} ± 0.01	0.02^{Aa} ± 0.01	0.04^{Ba} ± 0.01	0.08^{Ca} ± 0.01	0.02^{Aa} ± 0.01	0.04^{Ba} ± 0.01	0.08^{Ca} ± 0.01	0.02^{Aa} ± 0.01	0.04^{Ba} ± 0.01	0.08^{Ca} ± 0.01	0.02^{Aa} ± 0.01	0.04^{Ba} ± 0.01	0.08^{Ca} ± 0.01
Δ^8-THC	ND^{Aa}	ND^{Aa}	ND^{Aa}	ND^{Aa}	ND^{Aa}	ND^{Aa}	ND^{Aa}	ND^{Aa}	ND^{Aa}	ND^{Aa}	ND^{Aa}	ND^{Aa}	ND^{Aa}	ND^{Aa}	ND^{Aa}	ND^{Aa}	ND^{Aa}	ND^{Aa}
CBN	0.007^{Ab} ± 0.001	0.013^{Bb} ± 0.001	0.026^{Cb} ± 0.001	0.02^{Ac} ± 0.01	0.02^{Ac} ± 0.01	0.03^{Bc} ± 0.01	0.007^{Ab} ± 0.001	0.013^{Bb} ± 0.001	0.026^{Cb} ± 0.002	0.007^{Ab} ± 0.001	0.013^{Bb} ± 0.001	0.026^{Cb} ± 0.002	ND^{Aa}	0.013^{Bb} ± 0.001	0.026^{Cb} ± 0.003	ND^{Aa}	0.012^{Ba} ± 0.001	0.024^{Ca} ± 0.001

CBNA	$0.02^{Aa} \pm 0.01$	$0.03^{ABa} \pm 0.01$	$0.06^{Ca} \pm 0.01$	$0.02^{Aa} \pm 0.01$	$0.03^{ABa} \pm 0.01$	$0.06^{Ca} \pm 0.01$	$0.02^{Aa} \pm 0.01$	$0.03^{ABa} \pm 0.01$	$0.06^{Ca} \pm 0.01$	$0.02^{Aa} \pm 0.01$	$0.03^{ABa} \pm 0.01$	$0.06^{Ca} \pm 0.01$	$0.02^{Aa} \pm 0.01$	$0.03^{ABa} \pm 0.01$	$0.06^{Ca} \pm 0.01$	$0.02^{Aa} \pm 0.01$	$0.03^{ABa} \pm 0.01$	$0.06^{Ca} \pm 0.01$
CBL	ND^{Aa}	ND^{Aa}	ND^{Aa}	$0.01^{Bb} \pm 0.01$	$0.01^{Bb} \pm 0.01$	$0.02^{BCb} \pm 0.01$	ND^{Aa}	ND^{Aa}	ND^{Aa}	ND^{Aa}	ND^{Aa}	ND^{Aa}	ND^{Aa}	ND^{Aa}	ND^{Aa}	ND^{Aa}	ND^{Aa}	ND^{Aa}
CBLA	$0.013^{Aa} \pm 0.001$	$0.03^{Bb} \pm 0.01$	$0.05^{Cc} \pm 0.01$	$0.01^{Aa} \pm 0.01$	$0.02^{ABab} \pm 0.01$	$0.04^{Cc} \pm 0.01$	ND^{Aa}	ND^{Aa}	ND^{Aa}	ND^{Aa}	ND^{Aa}	ND^{Aa}	ND^{Aa}	ND^{Aa}	ND^{Aa}	ND^{Aa}	ND^{Aa}	ND^{Aa}
Sum of 17 cannabinoids	$5.53^{Ad} \pm 0.44$	$11.05^{Bd} \pm 0.88$	$22.08^{Cb} \pm 1.76$	$4.73^{Abc} \pm 0.46$	$9.21^{Bbc} \pm 0.90$	$16.81^{Cb} \pm 1.67$	$4.48^{Aab} \pm 0.44$	$8.48^{Bab} \pm 0.84$	$15.73^{Cab} \pm 1.58$	$4.27^{Aa} \pm 0.42$	$8.03^{Ba} \pm 0.80$	$15.22^{Ca} \pm 1.54$	$4.19^{Aa} \pm 0.41$	$7.81^{Ba} \pm 0.78$	$14.52^{Ca} \pm 1.48$	$4.03^{Aa} \pm 0.40$	$7.55^{Ba} \pm 0.76$	$14.11^{Ca} \pm 1.45$

a-f – the different small letters within the same row (in one amount of the input used, for example, 0.5%) indicate a significant differences (α <0.05) influenced by storage time; A-C – the different capital letters within the same row indicate significant differences (α <0.05) influenced by the differences between the content of the analyzed substance in each week of storage

Table S5. Cannabinoids determined in fermented milk drink with hemp oil input (0.5%, 1% and 2% (*w/v*)).

Analyte	Assumed amount of compound			After fermentation			1 week			2 weeks			3 weeks			4 weeks		
	**Amount in fermented milk drink (mg/100g of product)**																	
	0.5%	1%	2%	0.5%	1%	2%	0.5%	1%	2%	0.5%	1%	2%	0.5%	1%	2%	0.5%	1%	2%
CBD	21.29^{Aa} ± 1.50	42.57^{Ba} ± 2.98	85.14^{Ca} ± 5.96	42.22^{Ac} ± 0.39	46.22^{ABab} ± 0.50	111.78^{Cb} ± 2.55	27.32^{Ab} ± 1.70	57.35^{Bc} ± 2.30	118.18^{Cc} ± 3.22	42.74^{Ac} ± 3.12	61.95^{Bcd} ± 3.99	120.14^{Ccd} ± 3.21	45.69^{Ac} ± 4.13	74.12^{Be} ± 8.12	121.88^{Cd} ± 4.47	49.29^{Acd} ± 1.78	85.69^{Bf} ± 2.85	122.48^{Cd} ± 3.56
CBDA	3.93^{Ac} ± 0.28	7.86^{Bc} ± 0.55	15.72^{Cb} ± 1.10	0.86^{Aa} ± 0.02	1.26^{Ba} ± 0.03	13.44^{Ca} ± 2.17	2.33^{Ab} ± 0.72	4.16^{Bb} ± 0.51	16.16^{Cb}c ± 1.99	5.13^{Ad} ± 0.87	11.74^{Bd} ± 1.15	27.41^{Cd} ± 2.19	8.60^{Ae} ± 1.54	17.11^{Be} ± 0.99	28.96^{Cd} ± 4.74	9.03^{Af} ± 0.02	18.11^{Bef} ± 0.12	30.38^{Cd} ± 0.89
Δ^{9}-THC	0.09^{Ab} ± 0.01	0.17^{Bb} ± 0.01	0.35^{Cb} ± 0.02	0.03^{Aa} ± 0.01	0.04^{Ab} ± 0.01	0.05^{Ba} ± 0.03	0.03^{Aa} ± 0.01	0.04^{Ab} ± 0.01	0.05^{Ba} ± 0.03	0.03^{Aa} ± 0.01	0.04^{Ab} ± 0.01	0.05^{Ba} ± 0.03	0.03^{Aa} ± 0.01	0.04^{Ab} ± 0.01	0.05^{Ba} ± 0.03	0.03^{Aa} ± 0.01	0.04^{Ab} ± 0.01	0.05^{Ba} ± 0.03
Δ^{9}-THCA-A	0.14^{Ab} ± 0.01	0.28^{Bb} ± 0.02	0.55^{Cb} ± 0.03	0.01^{Aa} ± 0.01	0.01^{Aa} ± 0.01	0.02^{ABa} ± 0.02	0.01^{Aa} ± 0.01	0.01^{Aa} ± 0.01	0.02^{ABa} ± 0.02	0.01^{Aa} ± 0.01	0.01^{Aa} ± 0.01	0.02^{ABa} ± 0.02	0.01^{Aa} ± 0.01	0.01^{Aa} ± 0.01	0.02^{ABa} ± 0.02	0.01^{Aa} ± 0.01	0.01^{Aa} ± 0.01	0.02^{ABa} ± 0.02
CBC	0.08^{Aa} ± 0.01	0.16^{Ba} ± 0.01	0.33^{Cb} ± 0.01	0.18^{Ab} ± 0.01	0.20^{Bb} ± 0.01	0.89^{Cd} ± 0.10	0.19^{Ab} ± 0.02	0.20^{ABb} ± 0.01	0.25^{Ca} ± 0.02	0.06^{Aa} ± 0.01	0.31^{Bc} ± 0.01	0.31^{Bb} ± 0.01	0.18^{Ab} ± 0.02	0.39^{Bd} ± 0.02	0.49^{Cc} ± 0.03	0.34^{Ac} ± 0.01	0.61^{Be} ± 0.03	1.00^{Cd} ± 0.05
CBCA	0.15^{Aa} ± 0.01	0.30^{Ba} ± 0.02	0.60^{Ca} ± 0.04	0.28^{Ab} ± 0.01	0.37^{Bc} ± 0.02	1.26^{Cb} ± 0.03	0.37^{Bd} ± 0.02	0.33^{Ab} ± 0.01	1.18^{Cb} ± 0.22	0.31^{Ac} ± 0.03	0.43^{Bd} ± 0.07	1.21^{Cb} ± 0.11	0.28^{Ab} ± 0.02	0.49^{Bd}e ± 0.04	1.22^{Cb} ± 0.14	0.25^{Ab} ± 0.02	0.59^{Bf} ± 0.02	1.27^{Cb} ± 0.02
CBG	25.56^{Af} ± 1.78	51.11^{Be} ± 3.57	102.22^{Cf} ± 7.16	5.20^{Aa} ± 0.42	5.71^{Ba} ± 0.07	14.04^{Ca} ± 0.17	9.04^{Ab} ± 0.22	19.01^{Bb} ± 0.31	24.31^{Cb} ± 1.27	12.20^{Ac} ± 0.74	27.34^{Bc} ± 0.74	39.56^{Cc} ± 2.12	18.78^{Ad} ± 0.53	33.79^{Bd} ± 1.33	44.44^{Cd} ± 4.20	20.08^{Ae} ± 0.38	34.53^{Bd} ± 1.52	50.48^{Ce} ± 0.50
CBGA	0.06^{Aa} ± 0.01	0.12^{Ba} ± 0.01	0.30^{Ca} ± 0.01	0.14^{Ad} ± 0.01	0.20^{Bb} ± 0.01	0.67^{Cd} ± 0.02	0.13^{Ad} ± 0.01	0.20^{Bb} ± 0.02	0.64^{Cc} ± 0.02	0.11^{Ac} ± 0.02	0.21^{Bbb} ± 0.03	0.60^{Cc} ± 0.04	0.11^{Ac} ± 0.03	0.20^{Bb} ± 0.04	0.53^{Cb} ± 0.05	0.09^{Ab} ± 0.01	0.23^{Bc} ± 0.01	0.52^{Cb} ± 0.02
CBDV	0.07^{Aa} ± 0.01	0.14^{Ba} ± 0.01	0.28^{Ca} ± 0.02	0.15^{Ab} ± 0.01	0.19^{Bb} ± 0.01	0.70^{Cb} ± 0.02	0.16^{Ab} ± 0.02	0.25^{Bc} ± 0.04	0.70^{Cb} ± 0.03	0.16^{Ab} ± 0.02	0.29^{Bcd} ± 0.03	0.70^{Cb} ± 0.02	0.16^{Ab} ± 0.02	0.30^{Bd} ± 0.05	0.70^{Cb} ± 0.04	0.16^{Ab} ± 0.02	0.35^{Be} ± 0.02	0.70^{Cb} ± 0.03
CBDVA	0.07^{Aa} ± 0.01	0.14^{Bb} ± 0.01	0.27^{Ca} ± 0.02	0.17^{Ab} ± 0.01	0.30^{Bb} ± 0.01	0.75^{Cb} ± 0.02	0.17^{Ab} ± 0.02	0.30^{Bb} ± 0.04	0.75^{Cb} ± 0.03	0.17^{Ab} ± 0.02	0.32^{Bbc} ± 0.03	0.75^{Cb} ± 0.04	0.17^{Ab} ± 0.03	0.36^{Bb}c ± 0.04	0.78^{Cbc} ± 0.02	0.15^{Ab} ± 0.02	0.36^{Bbc} ± 0.02	0.78^{Cbc} ± 0.03
Δ^{9}-THCV	0.003^{Ab} ± 0.01	0.006^{Ba} ± 0.002	0.012^{Ca} ± 0.02	0.005^{Ac} ± 0.001	0.006^{Aa} ± 0.001	0.022^{Bc} ± 0.001	0.005^{Ac} ± 0.001	0.01^{Bb} ± 0.01	0.02^{Cb} ± 0.01	0.005^{Ac} ± 0.001	0.01^{Bb} ± 0.01	0.02^{BCb} ± 0.01	ND^{Aa}	0.01^{Bb} ± 0.01	0.02^{BCb} ± 0.01	ND^{Aa}	0.01^{Bb} ± 0.01	0.02^{BCb} ± 0.01
Δ^{9}-THCVA	0.007^{Aa} ± 0.001	0.01^{Ba} ± 0.01	0.03^{Ca} ± 0.0.1	0.015^{Ac} ± 0.001	0.022^{Bb} ± 0.001	0.06^{Cb} ± 0.012	0.02^{Ad} ± 0.01	0.03^{Bc} ± 0.01	0.07^{Cc} ± 0.010	0.02^{Ad} ± 0.01	0.03^{Bc} ± 0.01	0.07^{Cc} ± 0.01	0.01^{Ab} ± 0.01	0.03^{Bc} ± 0.01	0.07^{Cc} ± 0.012	0.01^{Ab} ± 0.01	0.03^{Bc} ± 0.01	0.07^{Cc} ± 0.01
Δ^{8}-THC	ND^{Aa}	ND^{Aa}	ND^{Aa}	ND^{Aa}	ND^{Aa}	ND^{Aa}	ND^{Aa}	ND^{Aa}	ND^{Aa}	ND^{Aa}	ND^{Aa}	ND^{Aa}	ND^{Aa}	ND^{Aa}	ND^{Aa}	ND^{Aa}	ND^{Aa}	ND^{Aa}
CBN	0.5^{Ac} ± 0.01	1.0^{Bc} ± 0.01	2.0^{Cc} ± 0.01	0.03^{Ab} ± 0.01	0.04^{ABa} ± 0.01	0.11^{Ca} ± 0.01	0.02^{Aa} ± 0.01	0.06^{Bb} ± 0.01	0.11^{Ca} ± 0.01	0.02^{Aa} ± 0.01	0.06^{Bb} ± 0.01	0.11^{Ca} ± 0.01	0.02^{Aa} ± 0.01	0.06^{Bb} ± 0.01	0.11^{Ca} ± 0.01	0.02^{Aa} ± 0.01	0.06^{Bb} ± 0.01	0.12^{Cb} ± 0.01

CBNA	0.004^{Aa} ± 0.001	0.007^{Ba} ± 0.001	0.014^{Ca} ± 0.001	0.01^{Ae} ± 0.01	0.01^{Ab} ± 0.01	0.03^{Bb} ± 0.01	0.007^{Ab} ± 0.001	0.015^{Bc} ± 0.001	0.03^{Cb} ± 0.01	0.008^{Ac} ± 0.001	0.019^{Bd} ± 0.002	0.03^{Cb} ± 0.01	0.009^{Acd} ± 0.002	0.02^{Bd} ± 0.001	0.03^{Cb} ± 0.01	0.01^{Ae} ± 0.01	0.02^{ABd} ± 0.0.1	0.03^{Bb} ± 0.0.1
CBL	0.02^{Aa} ± 0.01	0.03^{Ba} ± 0.01	0.06^{Ca} ± 0.01	0.03^{Ab} ± 0.01	0.03^{Aa} ± 0.01	0.11^{Bb} ± 0.01	0.06^{Ac} ± 0.01	0.09^{Bb} ± 0.02	0.14^{Cc} ± 0.03	0.07^{Ad} ± 0.01	0.10^{Bc} ± 0.01	0.14^{Cc} ± 0.03	0.07^{Ad} ± 0.01	0.10^{Bc} ± 0.02	0.14^{Cc} ± 0.03	0.07^{Ad} ± 0.01	0.10^{Bc} ± 0.01	0.20^{Cd} ± 0.01
CBLA	0.03^{Aa} ±0.01	0.05^{Ba} ± 0.01	0.10^{Ca} ± 0.01	0.05^{Ab} ± 0.01	0.06^{Bb} ± 0.01	0.25^{Cb} ± 0.02	0.05^{Ab} ± 0.01	0.10^{Bc} ± 0.01	0.25^{Cb} ± 0.02	0.05^{Ab} ± 0.01	0.10^{Bc} ± 0.01	0.25^{Cb} ± 0.02	0.05^{Ab} ± 0.01	0.10^{Bc} ± 0.01	0.25^{Cb} ± 0.02	0.05^{Ab} ± 0.01	0.11^{Bd} ± 0.01	0.26^{Cb} ± 0.02
Sum of 17 cannabinoids	52.00^{Abc} ± 4.68	103.95^{Bc} ± 9.36	208.10^{Cc} ± 18.72	49.38^{Ab} ± 4.48	54.67^{ABa} 4.97	±144.27^{Ca} ± 13.19	39.91^{Aa} ± 3.63	82.16^{Bb} ± 7.46	162.86^{Cab} ± 14.86	61.10^{Ad} ± 5.54	102.96^{Bc} ± 9.35	191.37^{Cbc} ± 17.44	74.17^{Ae} ± 6.72	127.13^{Bd} ± 11.54	199.69^{Cc} ± 18.20	79.59^{Aef} ± 7.21	140.85^{Be} ± 12.78	208.38^{Ccd} ± 18.99

a-f – the different small letters within the same row (in one amount of the input used, for example, 0.5%) indicate a significant differences (α <0.05) influenced by storage time; A-C – the different capital letters within the same row indicate significant differences (α <0.05) influenced by the differences between the content of the analyzed substance in each week of storage

Table S6. Sensory quality determinants used in the profile evaluation of samples with hemp input

No.	Determinants	Definition of sensory quality determinants	Scale markings
1.	Green colour	Green colour intensity	Light green - dark green
2.	Colour typical for yoghurt	Typical of a fermented milk baverage	typical - non-typical
3.	Yoghurt aroma	Characteristic of natural yoghurt	Undetectable - very intense
4.	Sour aroma	Characteristic of natural yoghurt	Undetectable - very intense
5.	Sweet aroma	Characteristic of natural yoghurt	Undetectable - very intense
6.	Aroma foreign characteristic 'grassy"	Foreign aroma, unusual for yoghurt	Undetectable - very intense
7.	Sweet flavour	Basic taste, no need for explanation	Undetectable - very intense
8.	Flavour foreign characteristic "grassy"	Foreign flavour, unusual for yoghurt	Undetectable - very intense
9.	Sour flavour	Acidic aftertaste, persists in the mouth	Undetectable - very intense
10.	Acidic flavour	Characteristic of natural yoghurt	Undetectable - very intense
11.	Bitter flavour	Basic taste, no need for explanation	Undetectable - very intense
12.	Consistency	Sensory sensation perceived upon ingestion of the sample	Smooth - perceptible elements
13.	Density	Sensory impression received when taking and consuming a sample	Very thin-very thick
14.	Overall quality	General sensory impression, including distinguishing marks of smell, colour, texture, taste	Bad - very good

Table S7. Results of the profile analysis of hemp-infused fermented milk drinks (0-10 scale).

No.	Determinants of aroma, colour, texture, taste and overall assessment	Control	Sample with hemp extract 0.5%	Sample with hemp extract 1%	Sample with hemp extract 2%	Sample with dried hemp 0.5%	Sample with dried hemp 1%	Sample with dried hemp 2%	Sample with hemp oil 0.5%	Sample with hemp oil 1%	Sample with hemp oil 2%
1.	Green colour	$0.01^{a} \pm 0.01$	$0.50^{b} \pm 0.09$	$1.33^{c} \pm 0.15$	$1.59^{cd} \pm 0.11$	$4.48^{e} \pm 0.54$	$6.74^{f} \pm 0.22$	$8.51^{g} \pm 0.45$	$0.01^{a} \pm 0.01$	$0.01^{a} \pm 0.01$	$0.01^{a} \pm 0.01$
2.	Colour typical for yoghurt	$0.01^{a} \pm 0.01$	$1.11^{b} \pm 0.12$	$2.25^{c} \pm 0.16$	$3.13^{d} \pm 0.40$	$8.24^{gh} \pm 0.42$	$8.85^{h} \pm 0.87$	9.35i ± 0.55	5.54e ± 0.19	$6.66^{f} \pm 0.45$	$7.42^{g} \pm 0.78$
3.	Yoghurt aroma	$9.59^{de} \pm 0.54$	$7.14^{b} \pm 0.41$	$7.22^{b} \pm 0.19$	$7.87^{bc} \pm 0.53$	$6.52^{a} \pm 0.47$	$6.89^{ab} \pm 0.69$	$7.12^{b} \pm 0.45$	$8.11^{c} \pm 0.24$	$8.58^{cd} \pm 0.45$	$8.98^{d} \pm 0.74$
4.	Sour aroma	$1.10^{a} \pm 0.14$	$2.16^{b} \pm 0.13$	$3.03^{c} \pm 0.20$	$3.89^{d} \pm 0.11$	$4.02^{e} \pm 0.14$	$5.37^{f} \pm 0.45$	$6.05^{h} \pm 0.24$	$5.23^{f} \pm 0.35$	$5.54^{fg} \pm 0.13$	$5.77^{gh} \pm 0.23$
5.	Sweet aroma	$5.74^{d} \pm 0.22$	$6.56^{e} \pm 0.20$	$4.44^{c} \pm 0.11$	$3.01^{b} \pm 0.01$	$2.06^{a} \pm 0.10$	$2.08^{a} \pm 0.12$	$2.19^{a} \pm 0.12$	$4.34^{c} \pm 0.21$	$4.38^{c} \pm 0.12$	$4.39^{c} \pm 0.14$
6.	Aroma foreign characteristic 'grassy"	$0.01^{a} \pm 0.01$	$0.03^{b} \pm 0.01$	$0.06^{c} \pm 0.01$	$0.09^{d} \pm 0.01$	$3.58^{f} \pm 0.14$	$5.74^{h} \pm 0.34$	$9.64^{j} \pm 0.45$	$3.04^{e} \pm 0.11$	$5.12^{g} \pm 0.14$	$7.85^{i} \pm 0.16$
7.	Sweet flavour	$5.58^{c} \pm 0.45$	$5.63^{c} \pm 0.12$	$5.54^{c} \pm 0.18$	$4.07^{b} \pm 0.13$	$2.03^{a} \pm 0.12$	$2.10^{a} \pm 0.18$	$1.99^{a} \pm 0.12$	$2.05^{a} \pm 0.14$	$2.09^{a} \pm 0.20$	$2.17^{a} \pm 0.16$
8.	Flavour foreign characteristic "grassy"	$0.01^{a} \pm 0.01$	$0.02^{a} \pm 0.01$	$0.07^{b} \pm 0.01$	$0.12^{c} \pm 0.02$	$3.31^{de} \pm 0.14$	$6.41^{g} \pm 0.15$	$9.48^{i} \pm 0.32$	$3.13^{d} \pm 0.09$	$5.35^{f} \pm 0.14$	$7.21^{h} \pm 0.17$
9.	Sour flavour	$0.01^{a} \pm 0.01$	$0.04^{b} \pm 0.01$	$1.41^{c} \pm 0.11$	$2.86^{de} \pm 0.45$	$4.14^{g} \pm 0.15$	$5.18^{h} \pm 0.21$	$6.36^{i} \pm 0.24$	$2.41^{d} \pm 0.12$	$2.58^{d} \pm 0.41$	$3.65^{f} \pm 0.11$
10.	Acidic flavour	$3.15^{a} \pm 0.67$	$4.51^{c} \pm 0.21$	$5.36^{d} \pm 0.14$	$6.06^{f} \pm 0.12$	$4.68^{c} \pm 0.22$	$5.45^{de} \pm 0.17$	$6.16^{f} \pm 0.41$	$4.12^{b} \pm 0.13$	$4.15^{b} \pm 0.11$	$5.14^{d} \pm 0.17$
11.	Bitter flavour	$0.01^{a} \pm 0.01$	$0.01^{a} \pm 0.01$	$0.05^{b} \pm 0.01$	$0.09^{b} \pm 0.01$	$2.58^{cd} \pm 0.19$	$5.35^{e} \pm 0.25$	$8.78^{f} \pm 0.11$	$1.99^{c} \pm 0.12$	$2.12^{c} \pm 0.17$	$2.22^{c} \pm 0.24$
12.	Consistency	$0.01^{a} \pm 0.01$	$1.00^{b} \pm 0.19$	$1.05^{b} \pm 0.01$	$1.40^{d} \pm 0.12$	$3.22^{e} \pm 0.07$	$7.13^{f} \pm 0.19$	$9.41^{g} \pm 0.52$	$1.01^{c} \pm 0.01$	$1.12^{c} \pm 0.14$	$1.18^{c} \pm 0.14$
13.	Density	$9.87^{f} \pm 0.21$	$5.47^{a} \pm 0.33$	$7.45^{bc} \pm 0.30$	$7.11^{b} \pm 0.11$	$8.01^{d} \pm 0.11$	$8.25^{de} \pm 0.41$	$9.65^{f} \pm 0.13$	$7.02^{b} \pm 0.14$	$8.14^{de} \pm 0.45$	$8.22^{de} \pm 0.16$
**14.**	**Overall quality**	$\mathbf{9.50^{i} \pm 0.11}$	$\mathbf{8.88^{gh} \pm 0.17}$	$\mathbf{7.57^{e} \pm 0.14}$	$\mathbf{7.04^{d} \pm 0.20}$	$\mathbf{5.12^{c} \pm 0.22}$	$\mathbf{4.78^{b} \pm 0.18}$	$\mathbf{3.36^{a} \pm 0.40}$	$\mathbf{8.12^{f} \pm 0.14}$	$\mathbf{8.14^{f} \pm 0.26}$	$\mathbf{8.35^{fg} \pm 0.40}$

a-i - the different small letters within the same row indicate a significant differences ($\alpha < 0.05$) influenced by amount and type of hemp input.
